# Acoustic-Lexical Characteristics of Child-Directed Speech Between 7 and 24 Months and Their Impact on Toddlers' Phonological Processing

**DOI:** 10.3389/fpsyg.2021.712647

**Published:** 2021-09-24

**Authors:** Margaret Cychosz, Jan R. Edwards, Nan Bernstein Ratner, Catherine Torrington Eaton, Rochelle S. Newman

**Affiliations:** ^1^Department of Hearing and Speech Sciences, University of Maryland, College Park, MD, United States; ^2^Center for Comparative and Evolutionary Biology of Hearing, College Park, MD, United States; ^3^Department of Communication Sciences and Disorders, University of Texas Health Science Center at San Antonio, San Antonio, TX, United States

**Keywords:** acoustics, lexicon, child-directed speech, phonological neighborhood density, speech clarity, phonological development, nonword repetition

## Abstract

Speech-language input from adult caregivers is a strong predictor of children's developmental outcomes. But the properties of this child-directed speech are not static over the first months or years of a child's life. This study assesses a large cohort of children and caregivers (*n* = 84) at 7, 10, 18, and 24 months to document (1) how a battery of phonetic, phonological, and lexical characteristics of child-directed speech changes in the first 2 years of life and (2) how input at these different stages predicts toddlers' phonological processing and vocabulary size at 2 years. Results show that most measures of child-directed speech do change as children age, and certain characteristics, like hyperarticulation, actually peak at 24 months. For language outcomes, children's phonological processing benefited from exposure to longer (in phonemes) words, more diverse word types, and enhanced coarticulation in their input. It is proposed that longer words in the input may stimulate children's phonological working memory development, while heightened coarticulation simultaneously introduces important sublexical cues and exposes them to challenging, naturalistic speech, leading to overall stronger phonological processing outcomes.

## 1. Introduction

The speech and language that children hear early in life is a strong predictor of their linguistic outcomes (Hart and Risley, 1995; Rowe, 2008; Huttenlocher et al., 2010). Children who are exposed to more child-directed speech (CDS) from caregivers eventually produce more complex babbling shapes, process speech faster, and grow larger vocabularies (Hoff, 2003; Weisleder and Fernald, 2013; Ferjan Ramírez et al., 2019). The slow speaking rate, dynamic pitch modulations, and shortened utterance lengths that characterize the unique CDS register are hypothesized to draw infants' attention and scaffold developmentally-appropriate linguistic models, facilitating speech-language development (Ferguson, 1977; Furrow et al., 1979; Fernald, 1985; Cooper and Aslin, 1990; Soderstrom, 2007; Wang et al., 2021). While the acoustic properties of CDS, and their changes over development, have been documented (Liu et al., 2009; Ko, 2012; Cristia, 2013; Hartman et al., 2017; Kalashnikova and Burnham, 2018), less is known about the phonological-lexical characteristics of CDS. This is a clear blindspot. For one thing, many of the acoustic measures previously studied, like vowel dispersion or speaking rate, covary with measures like word frequency and phonological neighborhood density. Consequently, previous observations about the effects of acoustic properties of CDS on children's speech-language outcomes could instead be attributable to lexical properties of the input. Furthermore, despite evidence that the quantity and quality of CDS predict a number of early phonological outcomes including babbling, infant speech perception, and toddler lexical processing—and suggestions in the literature that CDS quantity *should* impact children's phonological processing—no work to date has explicitly studied the relationship between CDS and children's phonological processing. This, again, leaves a critical gap in our understanding of development because different kinds of linguistic input may matter more, or less, to different developmental outcomes (e.g., lexical processing, speech discrimination) at different developmental stages. It is thus essential that we understand not simply *if* input matters for children's phonological processing, but also *how* and *when*.

To that end, this study has two goals. In a large cohort of English-learning children (*n* = 84), observed from 7 to 24 months, we first document longitudinal changes in a comprehensive battery of (North American) CDS characteristics. Then, in the same cohort, we model static CDS measures at 7, 10–11, and 18 months to predict toddlers' phonological processing and vocabulary sizes at 24 months. In doing so, we document how an array of co-varying lexical, phonological, and acoustic characteristics of CDS change over development, allowing us to disentangle and model how they contribute to toddlers' speech-language outcomes at 24 months.

## 2. Previous Literature

### 2.1. Changes in Child-Directed Speech With Age

Child-directed speech (CDS) is not a static construct over the first years of a child's life. From infancy through early toddlerhood, the mean length of CDS utterances increases (Stern et al., 1983), the vowel space and individual vowel categories expand and then contract (Bernstein Ratner 1984; Liu et al. 2009; Hartman et al. 2017, but see Burnham et al. 2015; Kalashnikova and Burnham 2018), and speaking rate increases (Ko, 2012; Sjons et al., 2017; Raneri et al., 2020). Fundamental frequency baselines and ranges also change non-linearly throughout this time (Stern et al., 1983; Kitamura et al., 2001; Kitamura and Burnham, 2003; Vosoughi and Roy, 2012; Han et al., 2018). While these studies demonstrate a fairly comprehensive understanding of age-related changes in the acoustics of CDS, our descriptions of phonological-lexical characteristics of CDS, as well as how they change over time, are more superficial. For example, it is well known that the number of word types, and frequency of rare words, in CDS increases as children age (Rowe, 2012; Glas et al., 2018). But there are far more sophisticated methods available to model the phonological-lexical properties of linguistic input. For example, sublexical organization of the lexicon can be modeled using phonological neighborhood density, or the number of words differing from a target word by one phoneme (e.g., the neighbors *sat* and *cat*). Words with many neighbors are said to reside in dense neighborhoods while those with few neighbors reside in sparse neighborhoods. Another related sub-lexical covariate is phonotactic probability, or the likelihood of a sound sequence in a language (e.g., *blick* is higher probability than *bnick* in English). Word frequency and word length (in phonemes) are likewise rarely included in models of CDS.

There are three reasons why it is important to model these phonological-lexical characteristics of CDS and their impact on children's speech-language outcomes. First, there is strong evidence that phonological-lexical properties of the input should impact children's phonological processing. Equivalent research has established such a relationship for children's word learning; for example, children learn words from dense neighborhoods first, followed by sparser neighborhoods (Storkel, 2004a; Storkel and Hoover, 2011; Kern and dos Santos, 2018; Zamuner and Thiessen, 2018). Second, the organization of the lexicon is reflected in *adult* speech production. Adults phonetically reduce (shorten segment durations, contract the vowel space, coarticulate more) in high-frequency relative to low-frequency words (Gahl, 2008; Bell et al., 2009), and in words from dense phonological neighborhoods relative to words from sparse neighborhoods (Scarborough, 2005; Gahl et al., 2012)^1^. Adults, and children, also reflect the structure of their lexicon in their speech as they produce high phonotactically probable sequences more smoothly (shorter durations) than low probability sequences (Edwards et al. 2004; see Vitevitch and Luce 2016 for overview). Adults are also known to reflect word structure in their speech. For example, adults compensate for prosodic structure via Compensatory Shortening, the phenomenon where word duration decreases as word length increases (Munhall et al., 1992; Harrington et al., 2015).

Finally, modeling characteristics of the lexicon, like phonological neighborhood density, in CDS is important because many of these measures are known to impact children's lexical access and processing. Dense phonological neighborhoods have been shown to inhibit lexical retrieval in children (Newman and German, 2002; Arnold et al., 2005), while high-frequency words, especially from sparse neighborhoods, are more rapidly recognized (Metsala, 1997)^2^. Evidence from the nonword repetition paradigm has also demonstrated that children process phonemically-shorter and phonotactically-probable words better than longer and/or less probable words (Gathercole et al., 1991; Edwards et al., 2004).

The conclusion from the above findings is that the acoustic properties of children's input, and the speed and accuracy of children's ensuing word recognition, vary systematically by word structure and the organization of the lexicon. Consequently, in development, it is not sufficient to only model CDS parameters like word types or tokens. Furthermore, and most critically, much work suggests that hyperarticulated CDS, like an expanded vowel space, may facilitate certain linguistic outcomes (Liu et al., 2003; Hartman et al., 2017). But as this body of research demonstrates, hyperarticulation (i.e., speech clarity) varies according to word structure and statistics: speakers hyperarticulate low-frequency words, phonetically reduce long words relative to short, etc. So the effects of these co-varying parameters, acoustic and lexical, need to be disentangled for children's development.

### 2.2. Nonword Repetition: An Important Indicator of Speech-Language Development

Phonological working memory, or the ability to recall sequences of phones, is a critical prerequisite to process—and thus learn—speech and language (Gathercole, 2006; Pierce et al., 2017). Children with stronger phonological working memories grow larger vocabularies (Adams and Gathercole, 1995; Baddeley et al., 1998), construct more complex syntactic constituents (Adams and Gathercole, 1995, 1996), and develop stronger phonological awareness skills (Michalczyk et al., 2013; Erskine et al., 2020).

Phonological working memory is often assessed using nonword repetition (NWR) tasks where participants process, temporarily store, and repeat novel, phonotactically-permissible sequences of phones (e.g., *blick*). NWR closely mimics novel word learning processes. In the task, children must not only activate phonological representations in response to an auditory stimulus, just as they do in, for example, looking-while-listening tasks, but they must also organize their speech motor-schemata to articulate a novel sequence of sounds. As such, NWR ability is unsurprisingly one of the strongest and most consistent predictors of children's future speech, language, and literacy development (Gathercole and Baddeley, 1989; Gathercole, 2006).

A variety of linguistic and experiential factors impact NWR accuracy, including stimulus length (Gathercole et al., 1991), phonological complexity (Szewczyk et al., 2018), and phonotactic probability/wordlikeness (Gathercole et al., 1991; Edwards et al., 2004; Szewczyk et al., 2018). Participants' vocabulary size (Gathercole and Baddeley, 1989; Munson et al., 2005; Hoff et al., 2008) and real-word repetition accuracy (Torrington Eaton et al., 2015) also predict NWR performance. Thus, although NWR was originally assumed to be a language-neutral diagnostic measure of phonological working memory, relevant experiential predictors, such as phonotactic probability, demonstrate that biologically-endowed working memory *and* experience with language, together, predict performance on the task (Gathercole and Baddeley, 1989; MacDonald and Christiansen, 2002).

One experiential predictor that developmental researchers have long assumed should predict children's NWR ability is linguistic input. There could be an indirect effect of input on NWR. Children who hear more CDS in their environments grow larger vocabularies (Hoff, 2003) and this vocabulary knowledge could result in a reorganization and early maturation of the lexicon, increasing NWR accuracy (Gathercole and Baddeley, 1989; Munson et al., 2005; Hoff et al., 2008).

Evidence for possible *direct* effects of language input on NWR, however, comes from a few different sources. First, studies show that children who receive more language input process speech faster during lexical identification tasks (Hurtado et al., 2008; Weisleder and Fernald, 2013), suggesting that there could be a similar relationship for NWR tasks. Elsewhere, in bilingual children, dual language exposure explains 20-25% of the variance in their NWR abilities at 22 months (Parra et al., 2011), meaning that bilingual children who are exposed to more of one of their languages have stronger NWR abilities in that language (though these exposure effects have not been found in older bilinguals; Core et al. 2017; Farabolini et al. 2021). Furthermore, children in an indigenous society with low reported rates of CDS were reported to have lower NWR scores than age-matched peers elsewhere in the literature (Cristia et al., 2020). And finally, a series of computational modeling studies that manipulated parameters of the input were able to replicate known developmental NWR patterns in 2- 6-year-olds, suggesting direct effects of input on NWR outcomes (Jones, 2016).

The assumption underlying all of this work is that children who are spoken to more, and engage in more conversations with caregivers, should have more practice processing incoming speech, encoding new words, and articulating novel sequences of phones—all skills that are implicated during NWR. However, despite these assumptions, and the strong evidence suggesting that such a link between input and phonological processing exists, to date no study has explicitly evaluated what parameters of the input predict children's NWR performance, a critical gap that the current study fills.

### 2.3. Research Questions

In sum, there are clear lexical (word types and tokens) and acoustic (vowel space, speaking rate) effects on children's speech-language development. However, many of these parameters vary systematically by the structure of the lexicon. The first goal of this paper is to document age-related changes in a battery of acoustic and lexical parameters of North American CDS. We ask:

1. How do organizational characteristics of the lexicon—phonological neighborhood density, word frequency, phonotactic frequency, word length—that are so predictive of adult speech production and children's lexical processing, change in CDS over development? Relatedly, how do frequently studied CDS measures, such as number of word types, change over development in this sample?

Then, to disentangle the effects of these co-varying acoustic and lexical parameters of children's input, we evaluate how each parameter predicts children's phonological processing and vocabulary sizes at 24 months.

2. What is the unique contribution of each acoustic and lexical input parameter, at 7, 10–11, and 18 months, for children's NWR and expressive vocabulary size at 24 months?

To answer these questions, we measure a host of acoustic and lexical parameters of CDS in semi-naturalistic caregiver-child interactions over the first 2 years of life as well as children's outcomes at 24 months.

## 3. Methods

### 3.1. Participants

Eighty-six mother-child pairs participated in the study. All children were born full-term, had normal hearing and vision, and heard primarily American English (approximately ≥85%) in the home at the time of initial recruitment (one child was also beginning to be exposed to Spanish at 7 months). Four children were in bilingual childcare settings at 24 months. All pairs were followed longitudinally from when the child was 7 to 24 months, and participated in a number of speech-language tasks including speech segmentation, phonological processing, and receptive and expressive vocabulary assessments, as well as free play sessions between the mother and child to elicit CDS samples.

Prior to analysis, two caregiver-child dyads were removed completely: one where the child did not complete the vocabulary assessments at 18 or 24 months and another where all of the transcripts of the mother-child interactions were unavailable for analysis. The gender distribution for the final sample of 84 participants was *n* = 49 female and *n* = 35 male children (see Table 1 for age information). Family socioeconomic status was quantified as mother's education level: 79 mothers (94%) had at least a college degree (1 did not respond). Caregivers identified their children's race/ethnicity as follows: 7 African American children (8.33%), 2 African American and white (2.38%), 3 Asian American and white (3.57%), 66 white (78.57%), 2 white Hispanic (2.38%), 3 non-white Hispanic (3.57%), and 1 child of mixed race and ethnicity. Forty-six (54.76%) of children were first-born, 32 (38.10 %) second-born, 4 (4.76%) third-born, and 1 each was the fourth- and fifth-born in the families.

**Table 1:** Child age and caregiver-child play session statistics.

**Timepoint**	**7mos**	**10–11mos**	**18mos**	**24mos**
Child age: mean (SD) range	7.5 (0.33) 6.93–8.27	10.5 (0.64) 8.77–12.13^*^	18.26 (0.57) 17.2–19.23	24.56 (0.57) 23.27–26.33
# of transcribed play sessions	82	83	40	83
# and % analyzed for coarticulation	81 (98.78)	82 (98.8)	40 (100)	83 (100)
# and % analyzed for vowels	75 (91.46)	78 (93.98)	40 (100)	82 (98.8)

* *Reflects child age (10 or 11 months) during collection of the CDS sample used in the analysis*.

An additional *n* = 39 children participated in the research program but either could not complete the NWR task (*n* = 30) or scored below the 10th percentile on the MacArthur-Bates Communicative Development Inventory (MB-CDI) (Fenson et al., 2007) at 24 months (*n* = 9); the data from these caregiver-child dyads are not analyzed here. We elected to remove the children who scored below the 10th percentile because that can be considered the cut-off for clinical diagnosis and it was not possible to ascertain diagnoses of language-related disorders (e.g., developmental language disorder) via other means given the children's young ages. See Torrington Eaton et al. (2015) for further details on participant exclusion.

### 3.2. Procedures

#### 3.2.1. Adult Language Samples

To elicit CDS samples, each caregiver-child pair participated in a free-play session in the lab at 7, 10, 11, and 24 months. Approximately half of the participants (*n* = 40, 47.62% of the sample) also completed a play session at 18 months. For the purposes of this study, the 10 and 11 month timepoint data were combined: *n* = 56 dyads (67.47% of the sample) contributed the CDS sample at 10 months and *n* = 27 (32.53%) contributed at 11 months.

During the play session, caregivers were instructed to interact and speak with their child naturally, as if they were at home. Participants were provided with a number of standardized toys and board books to ensure that a sufficient number of target vowels and segments were elicited over the course of the interaction. Caregivers were recorded with an Audio-Technica AT 8531 lavalier microphone connected to a Marantz PMD 660 solid-state recorder. Each session lasted between 15 and 20 minutes.

Two recordings, one at 11 months and another at 24 months, were removed because they were only approximately 5 minutes in length or shorter. An additional two 7-month recordings were removed due to poor audio quality/unavailability. Transcriptions from the caregiver-child play sessions in the lab can be found in the NewmanRatner corpus, available on CHILDES (https://childes.talkbank.org/access/Eng-NA/NewmanRatner.html) (MacWhinney, 2000; Newman et al., 2016).

#### 3.2.2. Word Repetition Tasks

At their 24-month visit, children completed a real word and corresponding nonword repetition task. Our goal in the NWR task was to evaluate phonological errors attributable to breakdowns in speech processing. However, 2-year-olds regularly make phonemic substitutions, due to ongoing articulatory maturation, that do not reflect their phonological processing skills. Consequently, we administered a real word repetition task, in addition to the nonword task, to control for children's articulatory skill during NWR (see section 3.3.4). To further ensure that we were evaluating children's phonological processing skills, and not their articulatory maturity, we also excluded late-emerging consonants such as /ɹ/ from the stimuli. See Torrington Eaton et al. (2015) for extensive modeling of these children's nonword and real word repetition results.

Stimuli for the real word and nonword repetition tasks consisted of *n* = 11 nonwords and *n* = 11 corresponding real words (*n* = 4 one-syllable, *n* = 4 two-syllable, and *n* = 3 three-syllable in each condition), matched for target consonants and consonant-vowel transitions by word condition (see Table A1 for stimuli list). Stimuli were adapted from Hoff et al. (2008). For the real word repetition task, children were handed small toys representing the target word and were prompted to repeat the word after the experimenter. For nonword task administration, the child was handed a brightly colored stuffed animal and the experimenter asked the child to repeat the “funny name.” The experimenter produced each item no more than two times before continuing to the next item. The real word repetition task was always administered before the nonword task to familiarize children with the task.

#### 3.2.3. Vocabulary Measurement

Children's vocabulary size was assessed at each timepoint in the longitudinal investigation. The MB-CDI (Fenson et al., 2007) was administered at 7, 10, 11 and 24 months for all children, and at 18 months for the *n* = 40 children tested at that timepoint (receptive vocabulary was assessed at ages 7, 10, and 11 months and expressive at 18 and 24 months). Additionally, the Peabody Picture Vocabulary Test, Fourth Edition (PPVT-4) (Dunn and Dunn, 2007) and Expressive One-Word Picture Vocabulary Test, 3rd edition (EOWPVT-3) (Brownell, 2000) were administered at 24 months. For the PPVT-4 we report raw scores because standard scores are only available for children older than 30 months.

### 3.3. Data Processing

#### 3.3.1. Cleaning Caregiver-Child Transcripts

From the caregiver-child transcripts, we excluded all onomatopoeia, exclamations (e.g., “ick!”), and proper names (except places likely to be common to all children in the sample such as “Maryland”), resulting in *n* = 3,463 word types across all timepoints and speakers. From these word types, contractions were excluded from the calculation of phono-lexical measures, as they are not included in the lexical statistics dictionary we used [*n* = 252 (7.28%) word types removed]; contractions were not excluded from the acoustic analysis.

#### 3.3.2. Measures of Phono-Lexical Diversity

A number of phono-lexical characteristics were calculated over transcripts of the caregiver-child interactions:

- Phonotactic Probability: probability of each word type in the transcript based on its average biphone positional probability in American English
- Phonological Neighborhood Density: number of phonological neighbors of each word type in the transcript
- Word Length: length, in phonemes, of each word type in the transcript
- Word Frequency: each word type's frequency in American English

We additionally computed the Type:Token Ratio (TTR) of each transcript, as well as the Mean Average Type:Token Ratio (MATTR) which is less sensitive to speech sample length than TTR (Fergadiotis et al., 2013). MATTR was computed over a 10-word token moving window (i.e., for a window of x tokens, MATTR is computed over tokens 1-x, 2-x, etc.). Finally, we computed the Type and Token count of each transcript. Many of these phono-lexical statistics are highly correlated, so they are evaluated separately for the statistical modeling.

The TTR, MATTR, Type count, and Token count were computed using Computerized Language ANalysis (CLAN) software program (MacWhinney, 2000). The lexical statistics were calculated using the Irvine Phonotactic Online Dictionary (IPhOD) (Vaden et al., 2009). The IPhOD computes phonotactic probability from biphone co-occurrence in English. Phonological neighborhood density statistics in the IPhOD were made according to Vitevitch and Luce (1999) and word frequency estimates in the dictionary were derived from the American English SUBTLEX database (Brysbaert and New, 2009). Word frequency and phonotactic frequency were log transformed prior to analysis. Following Storkel (2004b), phonotactic probability was additionally z-score normalized to control for word length confounds. For words with multiple pronunciation variants in the IPhOD, we selected the variant with the highest phonotactic probability. These lexical statistics are reported over word types, not tokens, within each speech sample, which is consistent with previous research.

Although estimates of neighborhood density and phonotactic probability based on children's lexicons are available (Storkel, 2004a), we elected to compute these measures over adult lexicons because our interest was in what components of *adult* speech best predicted children's phonological outcomes. Lexical statistics calculated over adult and child speech corpora are also strongly correlated (Guo et al., 2015).

#### 3.3.3. Acoustic Analysis

Given the large amount of acoustic data generated from 84 children, at multiple timepoints, we conducted the acoustic analysis over a subset—the second 5-min chunk—of each caregiver-child play session at 7, 10–11, and 24 months (excluding the 18-month sample since this was only collected from a subset of the dyads). The 5-min subsets of each play session were segmented into Praat TextGrids (Boersma and Weenink, 2020) and force-aligned to the phone level (McAuliffe et al., 2017). One of two trained phoneticians then hand-checked each TextGrid and adjusted the alignment as necessary. Because acoustic measures can be sensitive to segmentation, alignment was standardized in several ways. Periodicity in the waveform and formant structure in the spectrogram marked vowels. Vowels were distinguished from glides by the presence of a steady-state formant. In the absence of a steady-state formant, 50% of the segment was devoted to the glide and 50% to the vowel. Utterance-initial plosives were segmented at the start of their release. Nasals were identified by anti-formants and depressed intensities in the spectrogram and fricatives by high-frequency energy in the spectrogram and aperiodicity in the waveform. Speech that was whispered or yelled was removed from acoustic analysis as were all words whose spectral shape could not be deduced in the spectrogram due to phonetic reduction. Overlapping speech (i.e., with target child) was also marked to be excluded from analysis.

We computed four measures of hyperarticulation in the CDS samples: vowel dispersion, vowel space area, segment duration, and adjacent consonant-vowel/vowel-consonant coarticulation. To compute vowel dispersion and vowel space area, the first and second formant (F1 and F2) frequencies at the midpoint of the three peripheral /i, a, u/ vowels were measured using a triple formant tracker running Inverse Filter Control (Watanabe, 2001), Entropic Signal Processing System's “autocorrelation”, and Entropic Signal Processing System's “covariance” formant trackers (https://github.com/megseekosh/vocal_tract_vowel). Then, the median F1 and F2 from the three trackers were computed. Formant measurements were Lobanov-normalized to account for speaker-specific anatomical differences (Lobanov, 1971); all vowel results were replicated with unnormalized data as well, except where noted. The caregiver's average vowel space area was measured, at each timepoint, using the phonR package in R (see McCloy 2016 for detail on measurement technique). Finally, the *dispersion* of each vowel token (calculated from word types to avoid data skew due to high-frequency words within the transcript) was calculated as the distance of each vowel token along F1 and F2 from each speaker's median F1 and F2 values.

We implemented coarticulation as the acoustic distance between adjacent phones, using a custom Python script running Librosa packages (McFee et al. 2015; see Gerosa et al. 2006; Cychosz et al. 2019 for further details). Specifically, Mel-frequency log-magnitude spectra were averaged over the entirety of each target phone; coarticulation was then the Euclidean distance between the averaged spectra of neighboring phones. We did not compute coarticulation within (1) stop-vowel sequences because it was not possible to delimit the closure portion of utterance-initial stops or (2) voiceless glottal fricative-vowel sequences due to the weak spectral signature of those fricatives. Coarticulation was computed for all remaining consonant manners.

Because unstressed vowels are highly reduced in American English, the hyperarticulation measures involving vowels were only made over stressed vowels/sequences containing a stressed vowel (including if the vowel-consonant transcended a syllable boundary). We additionally only computed the hyperarticulation measures in content words, which is in keeping with previous work on the interaction of vowel space, coarticulation, and the lexicon (Gahl et al., 2012; Zellou and Scarborough, 2015).

We assessed changes in speaking rate by modeling segment duration, and not explicitly calculating maternal speaking rate as number of syllables/minute, for example, because speaking rate is highly correlated with segment duration and there have been recent reports on age-varying changes in maternal speaking rate in this corpus (Raneri et al., 2020).

#### 3.3.4. Nonword Repetition Scoring

The nonword stimuli contained *n* = 33 phoneme targets to be scored. To ensure that NWR errors were attributable to children's phonological processing, and not articulatory limitations, each phoneme produced in the NWR condition was compared to the equivalent phoneme in the real word condition. If the phoneme was produced incorrectly in both conditions, it was assumed to be attributable to articulatory limitations, and was not marked incorrect in the nonword condition. If the phoneme was produced incorrectly in only the nonword but not real-word condition, it was marked incorrect in the nonword condition. Nonwords that children failed to repeat after two experimenter prompts were also marked as inaccurate.

## 4. Results

Data were analyzed in the RStudio computing environment (version: 1.4.1103; RStudio Team 2020). Data visualizations were created with ggplot2 (Wickham, 2016). Modeling was conducted using the lme4 (Bates et al., 2015) and lmerTest (Kuznetsova et al., 2017) packages. Pairwise comparisons and model summaries were presented with emmeans (Lenth, 2021) and Stargazer (Hlavac, 2018). Model parameter significance was determined via a combination of log-likelihood comparisons between models, AIC estimations, and *p*-values from model summaries. Relevant variables were mean-centered prior to model fitting. All modeling and analysis scripts are included in the affiliated GitHub repository (https://github.com/megseekosh/cds-processing).

### 4.1. Age-Related Changes in Child-Directed Speech

**Figure 1:**
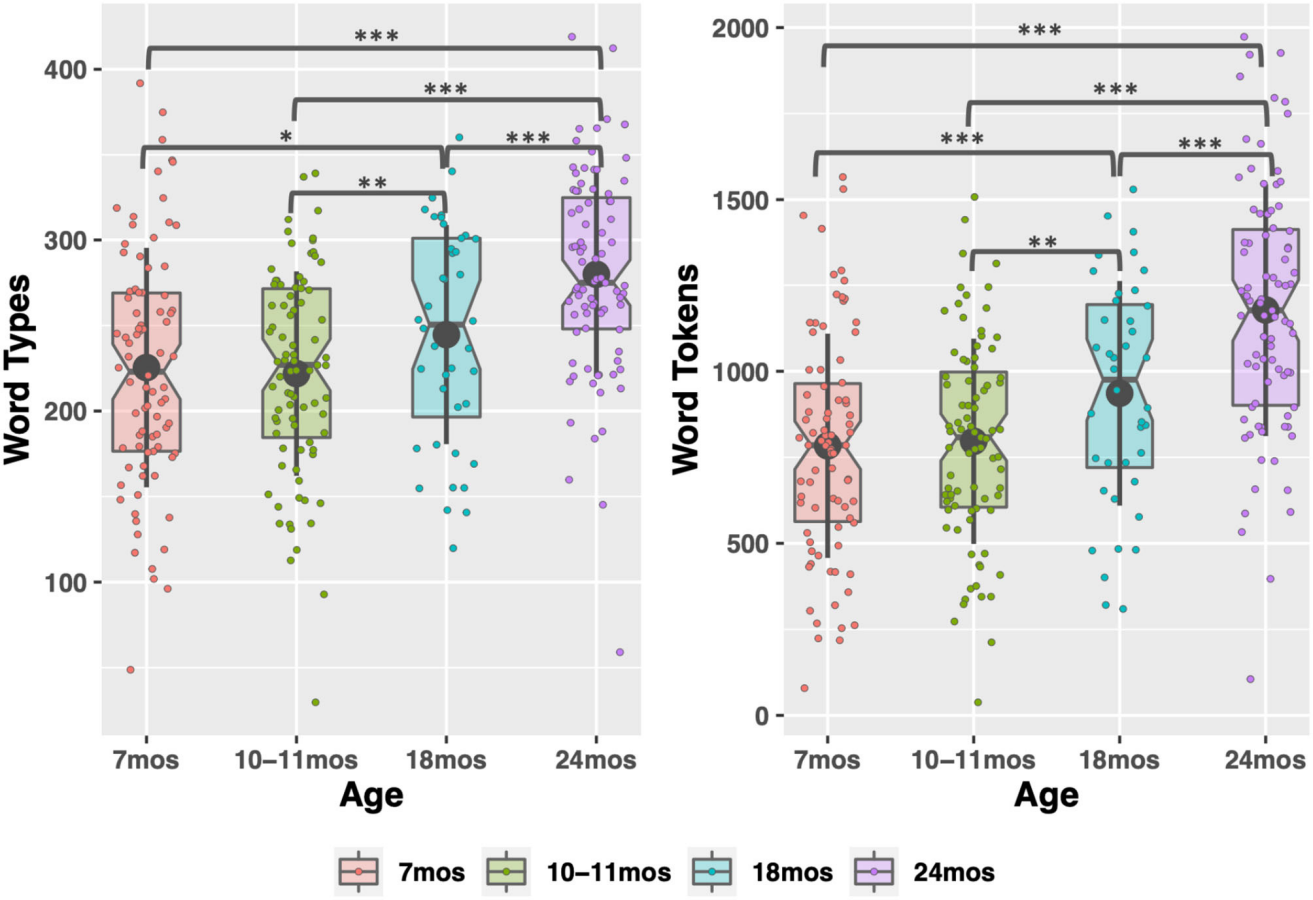
Word type and token count in child-directed speech between 7 and 24 months. Large, gray points indicate mean; whiskers indicate 1 SD from mean. Notches indicate median. *p < 0.05; **p < 0.01; ***p < 0.001.

**Figure 2:**
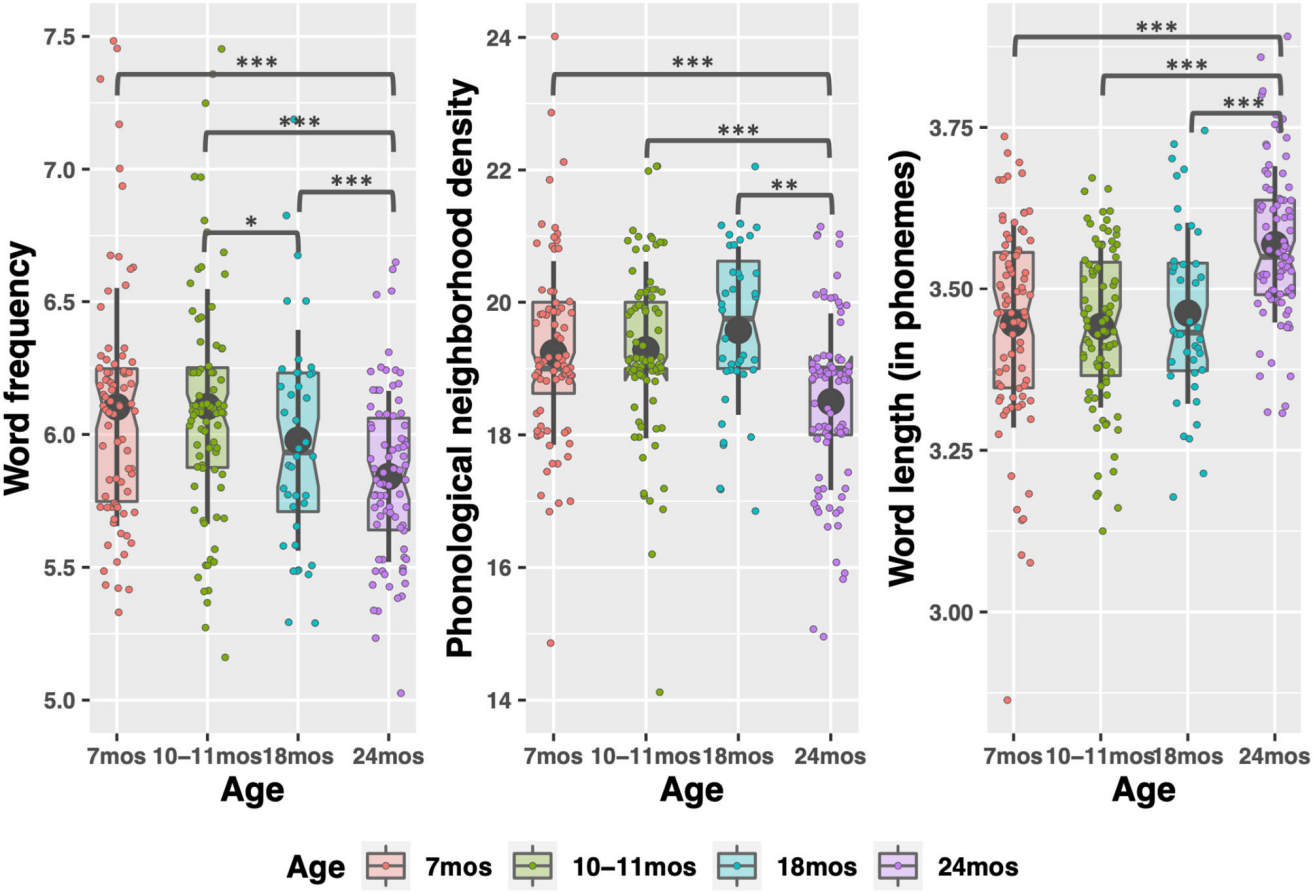
Phono-lexical characteristics of child-directed speech between 7 and 24 months. Large, gray points indicate mean; whiskers indicate 1 SD from mean. Notches indicate median. Individual datapoints indicate median word frequency and phonological neighborhood density (left and center figures), or mean word length (right figure). *p < 0.05; **p < 0.01; ***p < 0.001.

**Figure 3:**
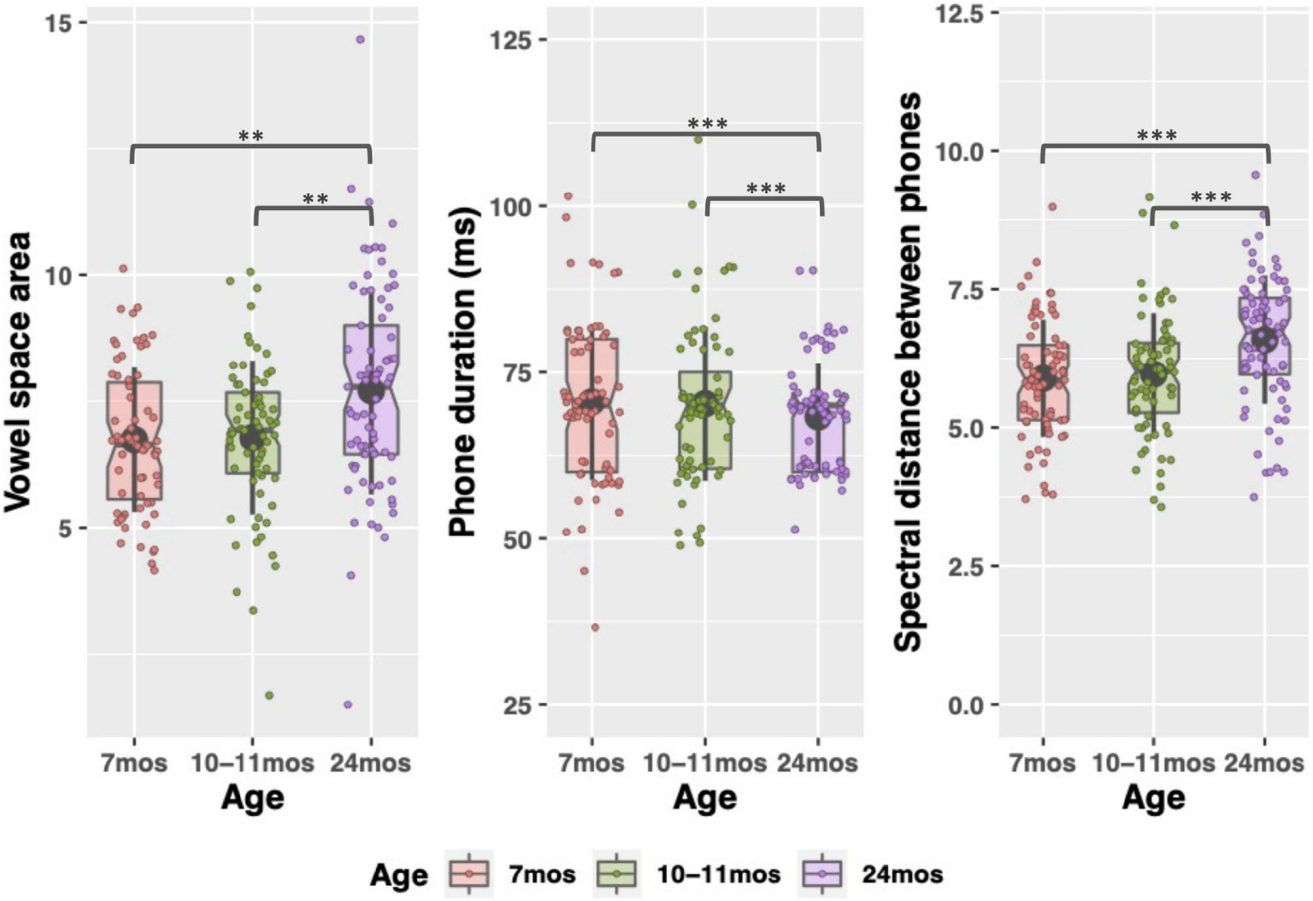
Acoustic characteristics of child-directed speech between 7 and 24 months. Large, gray points indicate mean; whiskers indicate 1 SD from mean. Notches indicate median. Individual datapoints indicate each caregivers' vowel space size (left figure), or median phone duration and coarticulation (center and right figures). *p < 0.05; **p < 0.01; ***p < 0.001.

Descriptive statistics for the acoustic-lexical CDS measures at 7, 10–11, 18, and 24 months are included in Table 2 and outlined in Figures 1–3). To evaluate these age-related changes in CDS, we fit a series of linear mixed effects models to predict each CDS measure. Each model included a random intercept of child-caregiver dyad and a fixed effect of timepoint.

**Table 2:** Descriptive statistics of child-directed speech characteristics at 7, 10–11, 18, and 24 months.

	**7mos**	**10–11mos**	**18mos**	**24mos**
	**Mean (SD) range**	**Mean (SD) range**	**Mean (SD) range**	**Mean (SD) range**
Types	246.63 (67.22) 49–392	237.76 (53.56) 30–339	261.28 (59.54) 120–360	292.68 (54.73) 59–419
Tokens	873.38 (313.16) 79–1,566	864.88 (277.02) 38–1,508	1,012.91 (302.73) 309–1,530	1,247.22 (346.29) 105–1,973
TTR	0.3 (0.06) 0.18–0.62	0.29 (0.06) 0.2–0.79	0.27 (0.05) 0.19–0.5	0.24 (0.04) 0.17–0.56
MATTR	0.88 (0.04) 0.78–0.95	0.87 (0.03) 0.74–0.95	0.89 (0.02) 0.83–0.93	0.91 (0.02) 0.82–0.95
Biphone probability	0.37 (1.73) −1.66–11.66	0.37 (1.71) −1.6–11.66	0.36 (1.7) −1.71–11.66	0.36 (1.71) −1.71–11.66
Word frequency	5.69 (2.56) −2.81–10.64	5.72 (2.53) −3.91–10.64	5.63 (2.53) −2.53–10.64	5.48 (2.57) −3.91–10.64
Phon. neighborhood density	20.55 (13.63) 0–50	20.65 (13.66) 0–50	20.56 (13.63) 0–50	19.94 (13.6) 0–50
Word length	3.48 (1.37) 1–12	3.47 (1.35) 1–12	3.49 (1.35) 1–14	3.59 (1.4) 1–13
Coarticulation (spectral distance)	6.89 (4.23) 0.93–34.75	6.91 (4.15) 1.04–36.91	NA	7.37 (4.23) 0.97–38.9
Phone duration (ms)	90.21 (70.78) 20.38–1,180	90.02 (72.73) 20.15–1,050	NA	83.45 (59.42) 20.13–880
Vowel space area	6.74 (1.43) 4.15–10.12	6.78 (1.52) 1.69–10.06	NA	7.7 (2.04) 1.5–14.65

There were significant effects of timepoint for all CDS measures except phonotactic probability, indicating that the input measures changed as children aged. Word type and token count increased significantly between each timepoint sampled, replicating previous work (Rowe, 2012), except 7 to 10–11 months (see Table S1 in the Supplementary Materials for pairwise comparisons of timepoints). The modeling also demonstrated how phono-lexical statistics of the input changed with child age. There was a significant, negative effect of the 24-month timepoint on the measures, indicating that children heard less frequent, longer words, from sparser neighborhoods, at 24 months than the other points (pairwise comparisons in Table S2 in Supplementary Materials).

Finally, there were significant changes in the acoustics between 7 and 24 months and 10–11 and 24 months where the speech became significantly faster (7–24 month changes: β = −7.32 *t* = −7.21 *p* < 0.001), but less coarticulated (7–24 month: β = 0.45, *t* = 5.94 *p* < 0.001) and produced with a more expanded vowel space (7–24 month: β = 0.99, *t* = 3.46, *p* = 0.002) (see Table S3 in Supplementary Materials for all pairwise comparisons). Given that vowels tend to reduce, and coarticulation increases, in faster speech, this pattern in the acoustics was somewhat surprising. However, as discussed in the introduction, many lexical statistics covary with acoustic properties so even as parents were speaking faster to their older children, the fact that they were using more diverse, lower-frequency words could explain the relative hyperarticulation in their speech at 24 months. (Again, acoustics were not measured at 18 months.)

In the descriptive statistics, one additional pattern emerged. Overall, the data trend is for CDS properties to resemble adult-directed speech more as children age. The exception to this is at 10–11 months, where many of the measures exhibit a hyper CDS register. There are, on average, fewer word types and tokens at 10 months than 7 months (the upper range of word count is also lower at 10 months). Words at 10 months tend to come from denser neighborhoods and be more coarticulated. We emphasize that the differences between 7 and 10–11 months are simply trends—no significant differences between the timepoints emerged in the modeling and there are no reliable differences between them. However, the trend suggests that parents may use a slightly more exaggerated CDS register at 10–11 months, as compared to just 3 months prior. We return to this point in the Discussion.

### 4.2. Modeling Relationships Between CDS, Phonological Processing, and Vocabulary Size

Having established that the quantity and quality of CDS speech differs by child age, we next evaluated how individual CDS differences explained the children's outcomes (NWR accuracy (i.e., phonological processing) and vocabulary size) at 24 months. Descriptive statistics of the children's outcomes are listed in Table 3, including the vocabulary measures at 7, 10, 11, and 18 months. Children varied greatly in performance on the NWR task (28–100% accuracy), and there was a similarly large range of vocabulary sizes at each timepoint sampled (i.e., 62–664 at 24 months). Expressive vocabulary size (MB-CDI) at 24 months is positively correlated with NWR accuracy at the same age (*r* = 0.26, *p* = 0.02), corroborating previous work on the relationship between the measures (Munson et al., 2005; Hoff et al., 2008)^3^.

**Table 3:** Child outcome measures at 7, 10, 11, 18, and 24 months.

	**7mos**	**10mos**	**11mos**	**18mos**	**24mos**
	**Mean (SD) range**	**Mean (SD) range**	**Mean (SD) range**	**Mean (SD) range**	**Mean (SD) range**
MB-CDI (receptive)^*^	9.55 (13.7) 0–83	41.81 (51.13) 0–359	62.49 (59.89) 1–331	NA	NA
MB-CDI (expressive)	NA	NA	NA	112.03 (108.6) 2–472	355.94 (150.32) 62–664
PPVT-4 (raw)	NA	NA	NA	NA	32.85 (12.36) 12–60
EOWVT (stan.)	NA	NA	NA	NA	97.94 (12.38) 55–118
Nonword rep. accuracy	NA	NA	NA	NA	0.65 (0.16) 0.28–1

* *MB-CDI measures receptive vocabulary from 7 to 11 months and expressive from 18 to 24 months*.

To model how the acoustic-lexical features of CDS predicted the children's outcomes, we fit a series of linear regression models outlining the relationship between input at the earlier stages—7, 10, and 18 months of age—on the children's outcomes at 24 months. Because there were different effects of acoustic and lexical CDS parameters by child age on NWR accuracy and vocabulary, we model acoustic and lexical parameters separately in the following sections.

#### 4.2.1. Modeling Predictors of Phonological Processing

Linear models were fit to predict each child's accuracy on the NWR task. To ensure that any effect of the CDS measures on NWR performance was attributable to the *input*, we needed to control for well-known baseline covariates of input (Maternal Education) and NWR (vocabulary size). Consequently, all NWR modeling includes these variables. In all cases, we modeled vocabulary concurrently with input since we wanted to control for the *predictive* nature of vocabulary for NWR and not simply its correlation with NWR at 24 months.

We next evaluated the role of each potential phono-lexical parameter: Word Frequency, Word Length (in phonemes), Phonological Neighborhood Density, Phonotactic Probability, Number of Word Types, Number of Word Tokens, MATTR, and TTR. Because the latter four variables are unnested, meaning they only provide one observation per transcript, while others are nested (i.e., each word type present in the transcript contributes an observation of phonotactic probability), we could not directly compare all of the variables in a straightforward manner. So we first present models of the nested variables, like Word Frequency, then the unnested variables, like Number of Word Types, and finally we propose a solution to model all phono-lexical parameters simultaneously.

In our modeling of nested lexical CDS parameters, we found effects of Word Length, Word Frequency, and Phonological Neighborhood Density in the CDS sample at 18 months on the children's NWR accuracy at 24 months. Children who heard longer words, less frequent words, and words from sparser neighborhoods, tended to have higher NWR accuracy. We were interested in the distinct contribution of each of these variables, but they are highly correlated (i.e., high-frequency words tend to be shorter) (Correlation matrices included in Supplementary Materials). So, to determine which correlated parameter(s) resulted in the best model fit, we regressed out each parameter's contribution to the model (Gahl et al., 2012). Specifically, our model-fitting procedure consisted of the following steps:

1. We fit a series of simple linear models predicting the role of each correlated parameter on the other. For example, we fit a model predicting the role of Word Frequency on Word Length. The resulting residuals from that model represented the contribution of Word Length not attributable to Word Frequency.
2. We included the calculated residuals and the ambiguous parameter (representing Word Frequency or Word Length) in linear models predicting outcomes like NWR or vocabulary.
3. We evaluated if the calculated residuals predicted the developmental outcome, above and beyond the ambiguous parameter.

**Figure 4:**
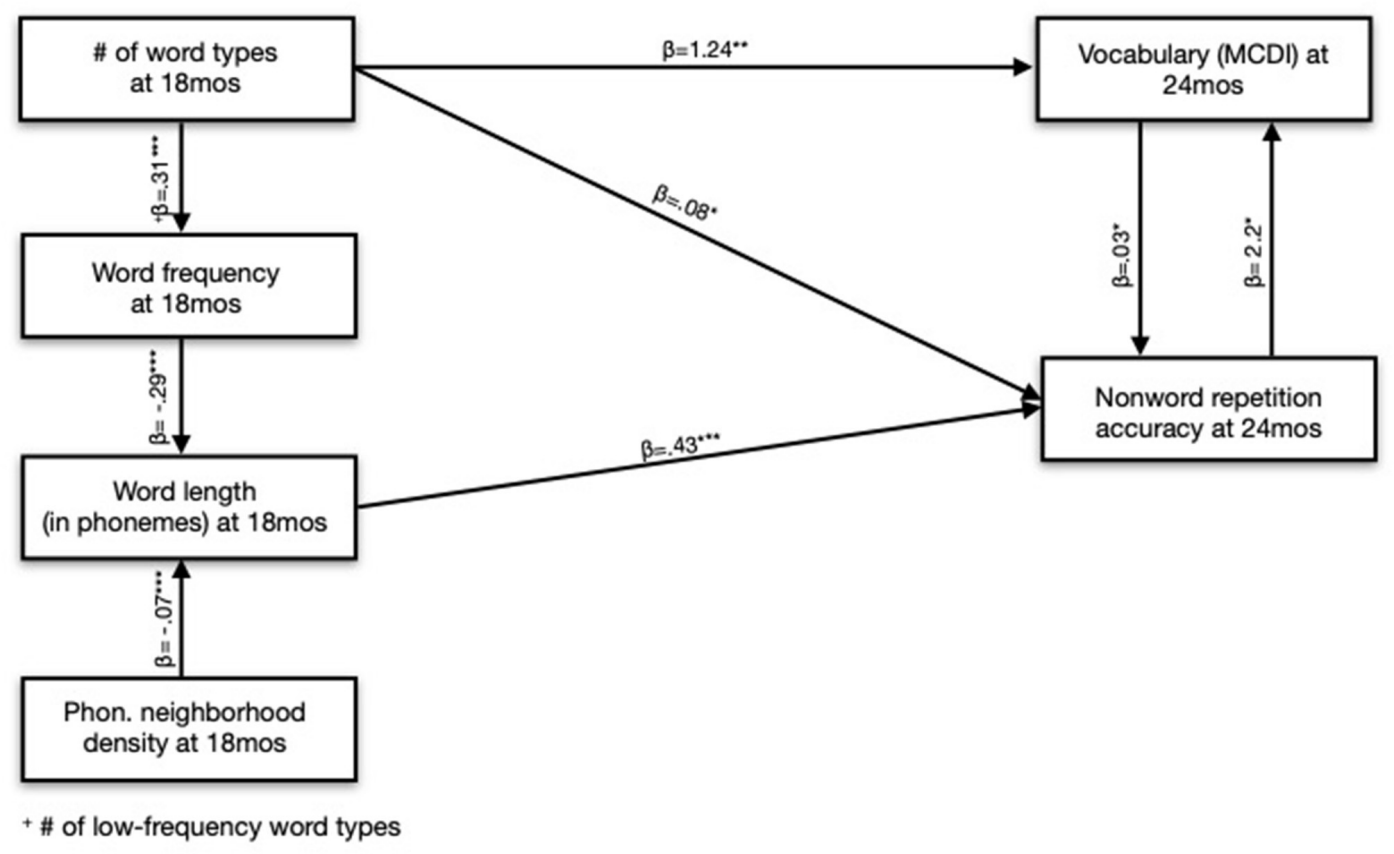
Lexical predictors at 18 months of nonword repetition accuracy and vocabulary size at 24 months.

In a model with Word Length residuals and Word Frequency (where Word Frequency could indicate either the role of Word Frequency *or* Word Length), we found that Word Length residuals predicted NWR accuracy. However, in a model with Word Frequency residuals and Word Length (where Word Length could indicate either the role of Word Frequency *or* Word Length), Word Frequency residuals did *not* predict NWR accuracy. From these results, we concluded that there was a direct effect of Word Length on NWR accuracy: children who heard longer words had stronger NWR skills. We also concluded that the observed effect of Word Frequency on NWR accuracy was indirect and explained by Word Length: children who heard less-frequent words demonstrated better phonological processing skills, but only because less-frequent words tend to be longer (in phonemes) (Figure 4 and Table 4).

**Table 4:** Modeling the effect of lexical CDS parameters at 18 months on nonword repetition at 24 months.

	**Word length**	**Word types**
	**Estimate^*p-value*^**	**Estimate^*p-value*^**
	**(95% CI)**	**(95% CI)**
Intercept	β = 43.36^***^	β = 22.14
	(42.15, 44.57)	(−3.30, 47.57)
	*t* = 70.31	*t* = 1.71
	*p* < 0.001	*p* = 0.10
Word length	β = 0.43^***^	
	(0.19, 0.66)	
	*t* = 3.55	
	*p* < 0.001	
Word types		β = 0.08^*^
		(0.004, 0.16)
		*t* = 2.06
		*p* = 0.05
Exp. vocab. (18 months)^†^	β = −0.03	β = −0.03
	(−0.03, −0.03)	(−0.07, 0.01)
		*t* = −1.30
		*p* = 0.21
Mat. Ed.	β = 7.38	β = 7.27^**^
	(7.05, 7.71)	(2.44, 12.10)
		*t* = 2.95
		*p* = 0.01
Observations	8,317	38
Residual Std. Error	14.79 (*df* = 8,313)	14.84 (*df* = 34)
F Statistic	704.19^***^ (*df* = 3; 8,313) (*p* < 0.001)	4.38^*^ (*df* = 3; 34) (*p* = 0.02)

* *p < 0.05*;

** *p < 0.01*;

*** *p < 0.001*.

† *In models containing unnested variables (Maternal Education and Vocabulary Size) and nested variables that are actually of interest (i.e., Word Length), alpha values and standard errors are artificially inflated so these statistics are not reported*.

We carried out a similar procedure to evaluate the relationship between Phonological Neighborhood Density and NWR accuracy. In a model with Word Length residuals and Phonological Neighborhood Density (where Phonological Neighborhood Density could indicate Phonological Neighborhood Density or Word Length), Word Length residuals predicted NWR accuracy. However, in a model with Phonological Neighborhood Density residuals and Word Length (where Word Length could indicate either parameter), Phonological Neighborhood Density residuals did *not* predict NWR accuracy. On the basis of this modeling, we also concluded that the effects of Phonological Neighborhood Density at 18 months were explained by Word Length. Again, children's phonological processing appears to benefit from hearing words from sparser neighborhoods, but this relationship is entirely explained by the fact that sparser words tend to be longer in length.

Lastly, we found effects of Word Length and Word Frequency at 7 months on NWR accuracy at 24 months, controlling for the children's receptive vocabulary size at 7 months. Following the same technique just outlined to regress out the contribution of the two correlated variables (Word Length and Word Frequency), we found that *both* Word Length and Word Frequency at 7 months predicted NWR at 24 months (see Supplementary Materials for visual and model summary). Altogether, however, the model of phono-lexical input at 18 months was a better fit to the NWR outcome.

We next assessed how the unnested lexical variables, like Word Token Count, predicted NWR outcomes. Only the parameter Number of Word Types at 18 months improved upon a model controlling for Maternal Education and the children's expressive vocabulary at 18 months: children who heard more word types at 18 months had higher NWR accuracy at 24 months. None of the remaining parameters (Word Tokens, TTR, MATTR) at any timepoint improved upon the baseline model.

To conclude the lexical modeling, we wanted to evaluate the contributions of Word Length and Number of Word Types at 18 months on NWR accuracy. When comparing nested and unnested independent variables such as these, researchers typically condense the nested variable to avoid overinflating the effect of the unnested variable (Foster-Johnson and Kromrey, 2018). One could, for example, model the average length of all word types and compare it to the number of word types. However, we assumed that caregivers did not necessarily differ in the average length of words in their speech. All speakers must use a large number of short, function words to communicate, resulting in little between-caregiver variability in a hypothetical parameter such as “average word frequency.” Instead, we hypothesized that caregivers would vary in the number of outlier observations—in this case, long words—in their speech.

With this idea in mind, we calculated the median word length of all word types uttered by all caregivers at 18 months. The median word length was four phonemes. Then, for each caregiver, we counted how many words they produced that were equal to or longer than (in phonemes) this median word length. The result of this calculation was a new unnested parameter that we created called Number of Long Words. Crucially, because Number of Long Words was unnested, we could directly compare it to Number of Word Types in a model predicting NWR outcomes.

**Figure 5:**
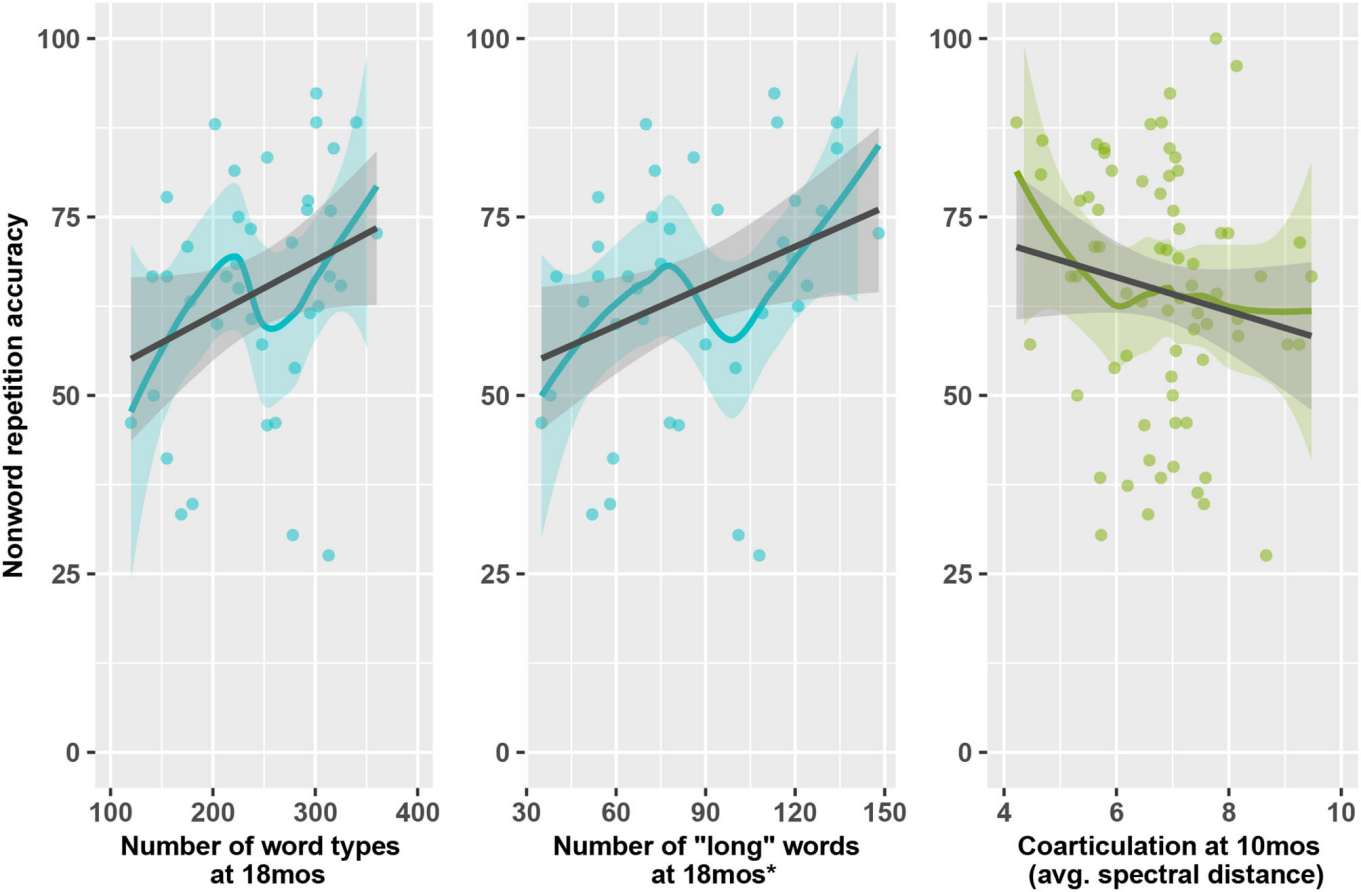
Acoustic-lexical predictors of nonword repetition at 24 months. On the left graph, increased spectral distance indicates less coarticulation. Gray regression represents model predictions. Colored points and local regressions represent original data. Ribbons represent 95% confidence intervals. *Word types containing 4+ phonemes.

Number of Long Words and Number of Word Types are necessarily correlated (the more distinct words you use, the longer your average word length). So, we regressed out the effect of each of these variables on the other to calculate residuals following the same method previously outlined. In a model predicting NWR outcomes, neither residuals for the parameter Number of Word Types nor Number of Long Words was significant. This result indicated that it was not possible to disentangle the effect of Number of Long Words from Number of Word Types on NWR accuracy: the modeling suggests that both variables, together, predict NWR accuracy (Figure 5).

Our final models predicting NWR evaluated the influence of acoustic CDS features: Vowel Space Size, Vowel Token Dispersion, Coarticulation, and Phone Duration. The effect of acoustic features was only apparent in the CDS sample from 10 to 11 months, not 7 months (acoustics were not measured at 18 months).

We found no effect of Vowel Space Size or Vowel Token Dispersion, at any time point, on the children's NWR outcomes, after controlling for Receptive Vocabulary at 11 months and Maternal Education^4^. However, both Coarticulation and Phone Duration at 10 months negatively predicted the children's NWR: children who heard slower, less coarticulated speech performed worse on the NWR task (Table 5).

**Table 5:** Modeling the effect of acoustic CDS parameters at 10–11 months on nonword repetition at 24 months.

	**Estimate^*p-value*^**
	**(95% CI)**
Intercept	β = 50.24^***^
	(48.05, 52.44)
	*t* = 44.88
	*p* < 0.001
Spectral distance	β = −0.17^**^
	(−0.28, −0.06)
	*t* = −2.97
	*p* = 0.004
Phone duration	β = −4.99
	(−13.81, 3.83)
	*t* = −1.11
	*p* = 0.27
Recep. vocab. (11 months)^†^	β = 0.02
	(0.01, 0.03)
Mat. Ed.	β = 3.69
	(3.13, 4.24)
Observations	5,241
Residual Std. Error	17.09 (*df* = 5,236)
F Statistic	50.72^***^ (*df* = 4; 5,236) (*p* < 0.001)

^*^p < 0.05;

** *p < 0.01*;

*** *p < 0.001*.

† *In models containing unnested variables (Maternal Education and Vocabulary Size) and nested variables that are actually of interest (i.e., Word Length), alpha values and standard errors are artificially inflated so these statistics are not reported*.

**Figure 6:**
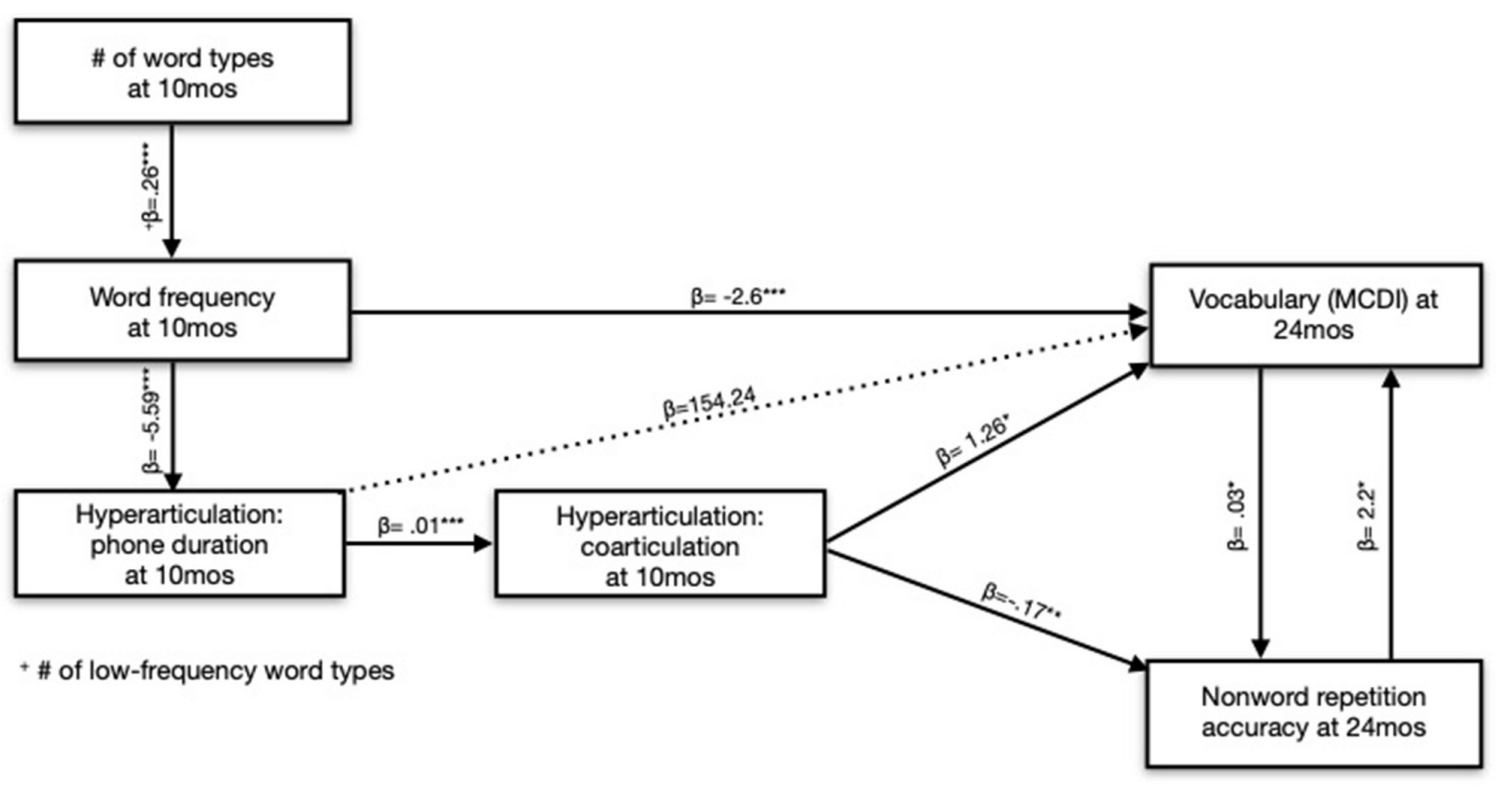
Acoustic predictors of nonword repetition accuracy and vocabulary size at 24 months.

We were interested in teasing apart the roles of Phone Duration and Coarticulation for NWR accuracy. However, the parameters are related because speakers tend to coarticulate more in faster speech (Gay, 1981). In our modeling of NWR accuracy, the best fit only included the variable Coarticulation (at 10 months), not Phone Duration. Adding Phone Duration to this model resulted in a slightly worse fit, and Phone Duration was not significant in the model summary. Nevertheless, we elected to include Phone Duration in the final model to control for the effect of speaking rate. Consequently, our Coarticulation parameter in the final model more accurately reflects the *unique* contribution of Coarticulation on NWR outcomes, controlling for speaking rate (Figure 6). Overall, however, we conclude that Coarticulation completely mediates the effect of Phone Duration on the children's NWR.

Additionally, although phonetic reduction, like coarticulation, is positively correlated with lexical statistics such as word frequency and word length (after controlling for frequency), we did not find effects of Word Length or Word Frequency at 10 months on the children's NWR outcomes. Thus, the effect of Coarticulation is not merely masking lexical effects such as Word Length: children who heard more coarticulated speech at 10 months performed better on the NWR task, irrespective of word length.

#### 4.2.2. Modeling Predictors of Expressive Vocabulary Size

In the final section, we modeled the effects of acoustic-lexical CDS parameters at 7, 10, and 18 months on expressive vocabulary size at 24 months. Linear models were fit to predict each child's reported expressive vocabulary size at 24 months. We again included baseline covariates known to predict children's vocabulary outcomes (Maternal Education and Child Gender): children of mothers with more years of education had larger vocabularies and girls had larger vocabularies than boys. All subsequent modeling includes these variables. As before, we first evaluate the nested lexical variables, then the unnested variables, and finally the acoustic parameters.

In a model predicting the children's expressive vocabulary size at 24 months, we found significant effects of Word Frequency and Word Length at 18 months. (We additionally found effects for these variables at 7 and 10 months, but the best model fit was again found for the measures at 18 months.) As before, we attempted to disentangle the effects of Word Frequency and Word Length by regressing the variables out. In doing so, we found effects of Word Frequency residuals in a model containing Word Frequency residuals and Word Length (where Word Length could indicate Word Frequency or Word Length), but no reliable effect of Word Length residuals in a model containing Word Length residuals and Word Frequency. This result indicates a direct effect of Word Frequency on vocabulary outcomes. Additionally, it shows that the effect of Word Length at 18 months on children's vocabulary outcomes at 24 months is entirely explained by Word Frequency: unsurprisingly, children who heard less frequent words tended to have larger vocabularies.

We next evaluated the contribution of unnested parameters like Number of Word Types. As anticipated from previous work, Number of Word Types, at 7, 10, and 18 months, significantly predicted the children's vocabulary sizes at 24 months: children who heard more diverse words went on to have larger vocabularies. Number of Word Tokens was likewise significant, but Number of Word Types provided the best model fit, indicating, as previous work has established (Rowe, 2012), that word diversity was of greater importance than raw word quantity for children's vocabulary development.

We next wanted to evaluate the contributions of Word Frequency and Number of Word Types at 18 months on children's vocabulary sizes at 24 months. To compare nested and unnested variables, we followed the same steps previously outlined: first, we calculated the median frequency of all word types produced by all caregivers at 18 months. Then, we calculated the number of words below the word frequency median that the caregiver produced. The result was an unnested parameter, Number of Low Frequency Words, that we compared to the unnested parameter Number of Word Types by regressing out the effect of each variable on the other and modeling the ensuing residuals.

We found a significant effect of Number of Word Types residuals in a model with those residuals and Number of Low Frequency Words. However, we did not find an effect of Number of Low Frequency Words residuals in a model with those residuals and Number of Word Types. This result led us to conclude that the Number of Word Types is the most relevant predictor of children's vocabulary outcomes: word frequency is only predictive in that if caregivers use more diverse words, they will, necessarily, eventually use words with lower statistical frequency in English (Figure 4 and Table 6).

**Table 6:** Modeling the effect of lexical CDS parameters at 18 months on expressive vocabulary at 24 months.

	**Estimate^*p-value*^**
	**(95% CI)**
Intercept	β = −67.15
	(−320.88, 186.58)
	*t* = −0.52
	*p* = 0.61
Word types	β = 1.24^**^
	(0.47, 2.02)
	*t* = 3.13
	*p* = 0.004
Gender: male	β = −75.80
	(−176.75, 25.15)
	*t* = −1.47
	*p* = 0.15
Mat. Ed.	β = 39.41
	(−8.89, 87.72)
	*t* = 1.60
	*p* = 0.12
Observations	40
Residual Std. Error	150.37 (*df* = 36)
F Statistic	4.41^**^ (*df* = 3; 36) (*p* = 0.01)

^*^p < 0.05;

** *p < 0.01*;

^***^p < 0.001.

As a final step in our modeling of vocabulary outcomes, we wanted to evaluate the contribution of the acoustic CDS parameters on the children's vocabulary outcomes. Unlike the NWR outcome, we found a significant, positive effect of increased, unnormalized Vowel Space Size at 10 months on expressive vocabulary size at 24 months (there was no effect of Vowel Token Dispersion at 7 or 10 months—the timepoints where acoustics were measured)^5^. However, an unnested model including only the parameter Word Type Count, as well as baseline covariates of Maternal Education and Gender, provided a better fit to the data so we conclude that Vowel Space Size is not a reliable predictor of vocabulary size in this dataset once lexical diversity of the input is considered.

We again found effects of Coarticulation and Phone Duration at 10 months on the expressive vocabulary outcomes. Specifically, children who heard slower, less coarticulated speech at 10 months had larger vocabularies at 24 months, controlling for Gender and Maternal Education, meaning that the direction of the effect of Coarticulation and Phone Duration differed by outcome (vocabulary vs. NWR). Again, speaking rate and coarticulation are correlated since speakers tend to coarticulate more in faster speech. Coarticulation improved upon a model with just Phone Duration, and both parameters were significant in the final model summary, leading us to conclude that both Phone Duration and Coarticulation, together, explained vocabulary outcomes. However, Coarticulation does, in part, mediate the effect of Phone Duration since (1) both parameters were significant in the model and (2) increased speaking rates *cause* increased coarticulation, but increased coarticulation does not cause speaking rates to increase (Table 7).

**Table 7:** Modeling the effect of acoustic-lexical CDS parameters at 10 months on expressive vocabulary at 24 months.

	**Word frequency**	**Coarticulation**
	**Estimate^*p-value*^**	**Estimate^*p-value*^**
	**(95% CI)**	**(95% CI)**
Intercept	β = 323.56^***^	β = 310.80^***^
	(313.01, 334.12)	(292.21, 329.39)
	*t* = 60.09	*t* = 32.77
	*p* < 0.001	*p* < 0.001
Word frequency	β = −2.60^***^	
	(−3.48, −1.72)	
	*t* = −5.81	
	*p* < 0.001	
Spectral distance		β = 1.26^*^
		(0.27, 2.26)
		*t* = 2.49
		*p* < 0.02
Phone duration		β = 154.24^***^
		(77.52, 230.96)
		*t* = 3.94
		*p* < 0.001
Gender: male	β = −54.57^***^	β = −60.16^***^
	(−59.08, −50.06)	(−68.32, −52.01)
	*t* = −23.74	*t* = −14.46
	*p* < 0.001	*p* < 0.001
Mat. Ed.	β = 17.89^***^	β = 22.50^***^
	(15.13, 20.64)	(17.67, 27.34)
	*t* = 12.72	*t* = 9.12
	*p* < 0.001	*p* < 0.001
Observations	16,214	5,255
Residual Std. Error	144.25 (*df* = 16,210)	148.55 (*df* = 5,250)
F Statistic	261.84^***^ (*df* = 3; 16,210) (*p* < 0.001)	78.91^***^ (*df* = 4; 5,250) (*p* < 0.001)

* *p < 0.05*;

^**^p < 0.01;

*** *p < 0.001*.

^†^*In models containing unnested variables (Maternal Education and Gender) and nested variables that are actually of interest (i.e. Word Frequency), alpha values and standard errors are artificially inflated, so these statistics are not reported*.

## 5. Discussion

Child-directed speech changes over the first years of a child's life, with ramifications for speech and language development (Stern et al., 1983; Huttenlocher et al., 2010; Ko, 2012; Hartman et al., 2017; Kalashnikova et al., 2018; Silvey et al., 2021). While age-related acoustic changes in CDS are well-documented, lexical statistics such as phonotactic probability and word frequency—which, crucially, are reflected in the acoustics of adult speech—are not. Consequently, the first goal of this paper was to document longitudinal changes in an exhaustive set of acoustic, phonological, and lexical characteristics in North American CDS between 7 and 24 months of age. Unsurprisingly, most of the CDS characteristics we measured did change as children aged. However, the measures did not necessarily progress over development in directions anticipated from previous work. Instead, we found that the most hyperarticulated speech occurred at 24 months, even as other characteristics of CDS became more adult-like, and we also observed a tendency for somewhat simplified CDS at 10–11 months.

### 5.1. Age-Related Changes in Child-Directed Speech

CDS is frequently described as a hyperarticulated speech register (Fernald, 2000), with classic theories arguing that the expanded vowel space enhances and clarifies acoustic categories (Kuhl et al., 1997). Hyperarticulation in CDS, along with other classic CDS characteristics such as a dynamic fundamental frequency, slower speaking rate, and shortened utterance length, is thought to reduce into an adult-directed speech register as children age. Yet caregivers here tended to hyperarticulate the most at 24 months, the oldest developmental stage observed, at a time when their speech might otherwise be expected to at least *start* resembling a more adult-directed register. There was also a trend—that did not emerge as significant in the modeling—for CDS characteristics to increase at 10–11 months relative to 7.

To a certain extent, North American caregivers are thought to modify parameters of their input, including the phonetics and phonology, to accommodate children's developing linguistic capacities (Snow, 1972; Gros-Louis et al., 2006; Leung et al., 2020). For example, caregivers' vowel spaces tend to expand as children start learning words (Dilley et al., 2014). So it is possible that the hyperarticulation we observed at 24 months stems from caregivers' implicit attempts to highlight phonological contrasts and elucidate individual segments in the input as their children are learning more words. However, we believe that the hyperarticulation at 24 months could have an additional source: the relationship between phono-lexical statistics and speech production. A coarse summary of the relationship between the structure of the lexicon and speech production is that phonetic reduction accompanies language use: short, probabilistic, and high-frequency words, from dense neighborhoods, tend to be phonetically reduced. And one defining characteristic that we observed of the CDS at 24 months was the overall use of longer, lower-frequency words, from sparser neighborhoods. It could thus be that the hyperarticulation observed at 24 months is not necessarily attributable to more extreme CDS at this timepoint or caregivers' implicit attempts to elucidate phonetic categories; rather, this hyperarticulation could reflect the statistically predictable properties of words that caregivers used when speaking to their children at that age.

An alternative explanation for the hyperarticulation at 24 months, and the trend for increased CDS at 10–11 months relative to 7, is that parents may only fine-tune aspects of their input after a certain developmental stage. A caregiver may assume that accommodation is unnecessary before their child has achieved certain levels of linguistic and conceptual maturity. And children's linguistic capacities, especially lexical and phonological, do change rapidly and noticeably over the time period sampled. At 7 months, typically-developing infants have just begun producing consonant-vowel transitions and reduplicated syllables (e.g., “bababa”) (Fagan, 2009). But by 10–11 months, a sizeable proportion of infants' vocalizations contain these transitions and reduplications, which are produced at increasingly faster speeds and with more fully-resonant vowels (Oller, 2000). Then, at 18 months, most infants have begun producing single, recognizable words and by 24 months their vocabularies are expanding rapidly during fast-mapping.

As caregivers are more likely to respond to infants' speech-like than non-speech-like vocalizations (Warlaumont et al., 2014), and to differentiate their feedback by the quality of infant vocalizations (Gros-Louis et al., 2006), we might expect input to differ between many of these timepoints. Specifically, we may observe hyperarticulation at 24 months, and a trend towards hyper CDS at 10–11 months, because caregivers could be engaging in cooperative communication (Renzi et al., 2017). They may recognize a need for linguistic accommodation to their infants at these ages thanks to, ironically, the infants' more advanced phonological and lexical capabilities and propensity to engage in contingent interaction compared to earlier timepoints.

Consequently, Goldilocks zones of infant phonological and lexical development—infants who are increasingly responsive and phonologically mature but not as linguistically advanced as young toddlers—may explain the hyperarticulation at 24 months and the trend toward hyper CDS (reduction in word type and token count, as well as neighborhood density and word frequency) at 10–11 months.

### 5.2. Language Input Drives Phonological Processing

The effects of language input on children's early lexical and morphosyntactic development have long been observed (Hoff, 2003; Huttenlocher et al., 2010; Bernstein Ratner, 2013; Weisleder and Fernald, 2013). Results concerning the role of input on phonological development, especially phonological processing and NWR, have been less conclusive. On the one hand, computational modeling and behavioral research on populations naturally-differing in input experience (bilingual children, cultures with low reported CDS rates) suggest that input *could* play a substantial role in some areas of speech development (Parra et al., 2011; Jones, 2016; Cristia et al., 2020). However, unlike other areas of language development, speech production interacts directly with the child's developing articulatory capabilities, potentially rendering production more immune to external factors such as adult input. Consequently, the second goal of this paper was to examine how the acoustic-lexical characteristics of CDS predicted children's NWR at 24 months. Given the strong, bi-directional relationships between children's NWR abilities and vocabulary sizes, we additionally modeled predictors of vocabulary growth. Overall, we found strong evidence for multi-faceted effects of input on NWR, suggesting that past null results could stem from the input measures assessed. The distinct effects of lexical diversity, word length, and hypoarticulation (coarticulation) on children's speech-language outcomes are addressed in the following sections.

#### 5.2.1. Lexical Diversity and Word Length Predict Phonological Processing

Lexical diversity in children's input, above and beyond quantity, results in stronger outcomes for just about every area of language development (Huttenlocher et al., 2010; Rowe, 2012). Here the relationship between lexical diversity and phonological processing/NWR could be explained as children who hear more word types from caregivers have more practice encountering, and potentially repeating, new words varying in phonological structure, length, and semantic content—skills relied upon during NWR. There are potentially additional, more indirect effects of lexical diversity on NWR as well. For example, children who are exposed to more diverse words in their input may also restructure their lexicons, including phonological neighborhoods, at a younger age relative to children who are repeatedly exposed to the same words (Charles-Luce and Luce, 1990; Storkel, 2004a). Among other effects, this lexical restructuring results in greater phonological awareness and phonological abstraction, allowing the children to repeat novel sequences of phones during the NWR task.

NWR ability is a key metric of phonological working memory (Gathercole, 2006; Pierce et al., 2017). Children who perform better on the task are better able to encode, remember, and articulate speech sounds. Modeling in this paper demonstrated that children who were exposed to longer words (in phonemes) performed better on the task, even after controlling for numerous variables, such as word frequency, that covary with word length. The effect of word length upon children's NWR accuracy could operate in the following manner: over time, children who are exposed to a higher modal word length in the input may hone their ability to remember sequences of phones—that is, their input provides them increased opportunity to develop their phonological working memories, a central component evoked during NWR.

Together, lexical diversity and word length dually contribute to NWR abilities at 24 months. To develop the skills required for NWR, children must be exposed to *diverse* words that allow them to practice novel word repetition and potentially restructure their lexicons. And children must also be exposed to somewhat *longer* words that exercise their phonological working memories. Lexical diversity and word length are still relatively coarse measures of the input. Both measures encompass a variety of constructs. For example, it could be not just the *length* of words in the input that promotes phonological processing but also the syllabic complexity of those words. We did not find effects of phonotactic probability, which may reflect phonological complexity to a certain extent, upon the children's outcomes. But our samples also showed little variability in phonotactic probability between or within speakers, so it could be that larger and/or more naturalistic samples would show effects of more detailed input measures such as phonological complexity or phonotactic probability upon children's phonological processing.

#### 5.2.2. Hypoarticulation Drives Phonological Processing; Hyperarticulation Drives Vocabulary Growth

The final predictor of children's speech-language ability at 24 months was the degree of coarticulation in the caregiver's speech at 10 months. An oft-repeated tenet in studies of CDS is that clearer speech leads to better linguistic outcomes, perhaps because CDS helps elucidate phonological categories and demarcates word boundaries, permitting syntactic bootstrapping (Gleitman, 1990). We did indeed find beneficial effects of clear speech at 10 months on children's vocabulary sizes: children of caregivers with more expanded vowel spaces, who spoke more slowly, and coarticulated less, grew larger vocabularies (the effect of vowel space size did not remain relevant after factoring in word type count, however). So, it was initially somewhat surprising to find a beneficial effect of *hypo*articulation, instantiated as increased coarticulation, upon children's phonological processing. We were, once again, able to control for a number of (though certainly not all) confounding variables, such as word frequency and speaking rate, that could otherwise explain the relationship between hypoarticulation and NWR. So the question remains: how does children's phonological processing benefit from hearing speech that is *more* coarticulated?

There are two mechanisms that potentially explain the beneficial effects of hypoarticulation upon phonological processing. First, it is important to clarify that coarticulation is more than random noise and variation in the speech signal. Rather, it provides important, contextual cues about word and segmental identity (Mann and Repp, 1980; Soli, 1981; Mattys et al., 2005; Gow and McMurray, 2007), facilitating word recognition in children as young as 18 months (Mahr et al., 2015). One principle of coarticulation is that it is largely planned (Whalen, 1990), meaning that speakers may subconciously manipulate variability in their speech to enhance communication, including to young children (Zellou and Scarborough, 2015). Thus, the first way that hypoarticulation drives phonological development is via the enhanced sublexical cues that maximally, naturalistically coarticulated input provides.

The other, complementary way that hypoarticulation could facilitate phonological processing outcomes may require reframing our assumptions about the developmental benefits of clear speech. It is often assumed that speech variability introduces noise, overlap, and confusion for infant and child learners, rather than an opportunity for children to scaffold into the adult speech stream. Relative to traditional CDS registers, adult-directed speech is phonetically reduced: it is spoken faster, resulting in more coarticulation and compromised phonological contrasts^6^. Consequently, children who receive more coarticulated speech in their input are exposed to highly confusable, overlapping speech categories, but they are also exposed to highly naturalistic speech exemplars that reflect a typical adult-directed speech register. Rather than a hindrance to development then, highly reduced, naturalistic speech—that nevertheless stems from a predictable source like the child's central caregiver—may prepare children to parse phonological units from a variety of speech registers, not just simplified CDS.

It is important to consider the developmental stages where we observed effects of hypoarticulation vs. word diversity and length upon the children's NWR accuracy. We did not measure the acoustics of CDS at 18 months, but we did not find a concurrent effect of coarticulation in caregiver speech at 24 months upon NWR at the same age. Nor did we find relationships between coarticulation in caregiver speech at 7 months and later NWR. While these null results cannot entirely rule out a role of hypoarticulation at 7 or 24 months—it could be that we didn't have sufficient word types to determine an effect at 7 months, for example—they do suggest that effects of hypoarticulation upon phonological processing outcomes may be limited to a certain developmental period. Why do we observe effects of coarticulation at 10 months, but not the other time periods? And why do we observe effects of lexical statistics, such as word type counts, at 7 and 18 months but *not* 10 months?

We believe these results demonstrate that, for phonological processing, it matters more *how* caregivers speak to 10–11 month-olds than the words they use. Parents who coarticulate more in the speech directed to their children are also speaking faster, thereby reducing their phonological contrasts, all factors that may be preparing their children to process and parse naturalistic speech. This more naturalistic input may even be preparing infants to benefit from overheard, adult-directed speech. It is obvious that a simplified CDS register, with its shortened utterances, isolated words, and longer pauses between utterances, helps infants break into the speech stream at, for example, 6.5–7.5 months (Nelson et al., 1989; Thiessen et al., 2005). Seven- to 8-month-old infants also have stronger lexical recognition and recall for words presented in an infant-directed register than an adult-directed register (Singh et al., 2009). Furthermore, in this study, we still found a clear speech benefit for the children's vocabulary outcomes. But conversely, after a certain point in development, children who are only exposed to easily-segmentable phonemes, syllables, and words may not develop the strongest phonological parsing abilities, making them less prepared to take advantage of more naturalistic, overheard and/or adult-directed speech in their environments.

Taken together, these three predictors of phonological processing—coarticulation, lexical diversity, and word length—suggest a complex, time-varying effect of input upon children's phonological processing outcomes. As such, it is not entirely surprising that previous work on this topic has proven inconclusive. For one thing, some effects of the input, such as word length, may be specific to certain phonological outcomes like NWR. As discussed above, the type of effect, acoustic vs. lexical, also appears to depend upon the timepoint studied.

Since we only sampled the children and their caregivers at discrete, non-random timepoints, we cannot definitively say that certain features (i.e., hypoarticulation) will always best stimulate phonological processing at certain developmental stages (i.e., 24 months). But these results may instead have some broader implications. Caregivers and early educators could consider modifying their speech-language patterns (speed, acoustic reduction, lexical diversity) in accordance with a child's developing linguistic capabilities, gradually increasing the prevalence of adult-directed speech characteristics as children age. Furthermore, there are many benefits of CDS beyond its slower speed and repetitiveness. Infants and children are also attracted to CDS registers because, relative to adult-directed speech, CDS is typified by greater pitch modulations (e.g., Kitamura et al. 2001), more eye-to-eye contact and positive affect between caregivers and children (Singh et al., 2002), and caregivers' exaggerated facial and bodily movements (Brand et al., 2002; Green et al., 2010). So adults could consider combining some aspects of adult-directed speech (e.g., faster speech rates, hypoarticulation) that scaffold the development of phonological processing skills with some aspects of CDS (e.g., positive affect, exaggerated facial expressions) that draw and maintain infants' and children's attention to the speech signal and conversational exchange.

Previous work on input in language development has been somewhat biased to certain outcome measures (vocabulary tests) and input measures (quantity and semantic quality of lexical items) because these are relatively straightforward measures to collect and compute. But a complete model of the role of input in development, one that predicts individual variability in speech production outcomes as well as more traditional measures such as vocabulary size or speech perception, clearly needs to incorporate a diverse set of acoustic and lexical parameters of the input, as this study has demonstrated.

### 5.3. Future Work

This work assessed children's input at 7, 10–11, 18, and 24 months and found time-varying CDS patterns with different effects on children's speech-language outcomes. Going forward, it will be important to sample input at additional, more regular time periods, particularly between 10–11 and 18 months. We cannot say, for example, if these age-related changes in CDS are linear or undergo additional changes at periods that were not observed.

Additionally, although our in-lab CDS samples allowed us to collect the high-quality audio required for the acoustic analysis, these play sessions likely do not entirely reflect typical caregiver-child interactions in the home. They are also of limited length (15–20 min). Some measures may be more biased than others by this sampling method. For example, while we believe that a 15–20 min interaction in the lab may reflect the diversity of word types typical of the caregiver's speech, this sampling strategy may not reflect word token count (and thus measures based on word token counts such as TTR and MATTR). Lexical, and especially phonetic, transcription is a lengthy, painstaking process, but going forward we should strive to collect high-quality acoustic samples of maximally-naturalistic CDS in the home to corroborate the results that we derived from the semi-naturalistic caregiver-child interactions in this paper.

## 6. Conclusions

The characteristics of child-directed speech (CDS) change over the first years of a child's life. Understanding how these changes unfold, and the consequences they have for children's speech-language development, is a key part of understanding the role of input for language development. We measured lexical, phonological, and acoustic properties of CDS at 7, 10–11, 18, and 24 months and found that the most significant changes in CDS occur in the second year of life. However, the developmental trend of CDS does not always progress to a more adult-directed speech register as children age. Rather, caregivers use a greater number and diversity of words at 24 months, increasing their use of low-frequency words, from sparser phonological neighborhoods, and driving hyperarticulation in their speech. Consequently, another source of hyperarticulation in CDS, beyond caregivers' implicit attempts to highlight phonological contrasts, may be lexical statistics.

We additionally measured how these properties of CDS predicted children's phonological processing and vocabulary at 24 months. Children's phonological processing benefited most from *hypo*articulation at 10 months, and longer, more diverse word types at 18 months, while vocabulary benefited from hyperarticulation and lexical diversity. Thus, novel measures of CDS, beyond lexical quantity and quality, demonstrated how language input could drive phonological development. Taken together, these results demonstrate how different characteristics of CDS vary by children's age, and how those characteristics promote speech-language development at distinct developmental stages.

## Data Availability Statement

The NewmanRatner corpus analyzed for this study can be found in the CHILDES component of Talkbank (https://childes.talkbank.org/access/Eng-NA/NewmanRatner.html).

## Ethics Statement

This study was approved by the University of Maryland, College Park Institutional Review Board. Participants provided written consent to participate.

## Author Contributions

RN and NB collected the data. MC, RN, and JE designed the research. CT scored the children's productions. MC analyzed the child-directed speech samples, conducting the modeling, and wrote the manuscript. All authors contributed to the article and approved the submitted version.

## Funding

This work was funded by National Institute on Deafness and Other Communication Disorders grant T32DC000046 to the University of Maryland (MC) and National Science Foundation grant BCS 0745412 (RN and NB).

## Conflict of Interest

The authors declare that the research was conducted in the absence of any commercial or financial relationships that could be construed as a potential conflict of interest.

## Publisher's Note

All claims expressed in this article are solely those of the authors and do not necessarily represent those of their affiliated organizations, or those of the publisher, the editors and the reviewers. Any product that may be evaluated in this article, or claim that may be made by its manufacturer, is not guaranteed or endorsed by the publisher.

## Footnotes

^1^Previous reports of greater dispersion in words from dense neighborhoods were generally due to confounds with lexical frequency which Gahl et al. (2012) were able to statistically control.

^2^Dense neighborhoods inhibit adults' lexical access and recognition as well (Luce and Pisoni, 1998; Vitevitch and Luce, 1998), but this is less relevant for the study at hand.

^3^We report on the relationship between NWR and concurrent vocabulary (24 months) measure because only approximately half of the children completed measures at 18 months.

^4^For the 10–11 month timepoint models, we modeled the children's vocabulary at 11 months, even though we had samples at 10 months as well, because 2 children were reported to not recognize any words at 10 months.

^5^There was no effect of normalized Vowel Space Size on vocabulary outcomes, leading us to hypothesize that previous reports on the developmental benefits of expanded vowel spaces could be attributable to an expanded f0 range, something that is controlled for in normalized vowel data.

^6^A faster speaking rate doesn't have to imply reduced speech intelligibility: with training, talkers can produce a clear speech register at a conversational speaking rate (Krause and Braida, 2004).

## A. Appendix

**Table A1:** Real word and non-word stimuli.

**Real word**	**Non-word (orthography)**	**Phonetic transcription**
Dog	kog	['kɔg]
Juice	buice	['bjus]
Cat	jat	['ʤæt]
Book	dook	['dʊk]
Balloon	challoon	[tʃɑ.'lun]
Cookie	pookie	['pʊ.ki]
Puppy	kuppy	['kʌ.pi]
Chicken	bicken	['bɪ.kən]
Banana	bajapop	[bɑ.'jæ.pəp]
Telephone	telina	[tɛ.'li.nɑ]
Lollipop	lolamas	['lɑ.lɑ.mɑs]
Pajamas	panaphone	[pə.'næ.foən]
