# Supplementary material for: Acoustic-Lexical Characteristics of Child-Directed Speech Between 7 and 24 Months and Their Impact on Toddlers' Phonological Processing
Source: Front Psychol. 2021 Sep 24;12:712647. doi: 10.3389/fpsyg.2021.712647 (PMC8497969; doi:10.3389/fpsyg.2021.712647)
Supplement: Supplementary file 1 [file Data_Sheet_1.pdf]

frontiers

Supplementary Material

1 SUPPLEMENTARY TABLES AND FIGURES

Table S1. Pairwise comparisons between timepoints for lexical statistic measures of CDS

Timepoints (months)	Estimate	S.E.	t-statistic	p-value	95% CI
**Tokens**					
7 - 10/11	-16.57	31.59	-0.52	0.953	-98.42 – 65.27
7 - 18	-175.54	40.99	-4.28	<.001	-281.7 – -69.39
7 - 24	-392.87	31.59	-12.44	<.001	-474.71 – -311.02
10/11 - 18	-158.97	40.94	-3.88	0.001	-265.01 – -52.93
10/11 - 24	-376.29	31.46	-11.96	<.001	-457.79 – -294.79
18 - 24	-217.32	40.94	-5.31	<.001	-323.36 – -111.28
**Types**					
7 - 10/11	3.05	6.07	0.50	0.958	-12.66 – 18.77
7 - 18	-22.21	7.87	-2.82	0.027	-42.59 – -1.82
7 - 24	-54.18	6.07	-8.93	<.001	-69.9 – -38.46
10/11 - 18	-25.26	7.86	-3.21	0.008	-45.63 – -4.9
10/11 - 24	-57.24	6.04	-9.47	<.001	-72.89 – -41.58
18 - 24	-31.98	7.86	-4.07	<.001	-52.34 – -11.61

Table S2. Pairwise comparisons between timepoints for phonological-lexical statistic measures of CDS

Timepoints (months)	Estimate	S.E.	t-statistic	p-value	95% CI
**Word Length**					
7 - 10/11	0.01	0.02	0.76	0.872	-0.03 – 0.05
7 - 18	-0.02	0.02	-0.86	0.824	-0.07 – 0.03
7 - 24	-0.11	0.01	-7.57	<.001	-0.15 – -0.07
10/11 - 18	-0.03	0.02	-1.47	0.457	-0.08 – 0.02
10/11 - 24	-0.12	0.01	-8.38	<.001	-0.16 – -0.08
18 - 24	-0.09	0.02	-5.02	<.001	-0.14 – -0.05
**Word Frequency**					
7 - 10/11	-0.03	0.03	-1.04	0.724	-0.1 – 0.04
7 - 18	0.07	0.04	1.95	0.206	-0.02 – 0.16
7 - 24	0.21	0.03	8.03	<.001	0.15 – 0.28
10/11 - 18	0.10	0.04	2.78	0.028	0.01 – 0.19
10/11 - 24	0.24	0.03	9.14	<.001	0.18 – 0.31
18 - 24	0.15	0.03	4.23	<.001	0.06 – 0.23
**Phon. Neigh. Density**					
7 - 10/11	-0.08	0.15	-0.54	0.949	-0.47 – 0.31
7 - 18	0.03	0.19	0.16	0.999	-0.45 – 0.51
7 - 24	0.62	0.14	4.36	<.001	0.26 – 0.99
10/11 - 18	0.11	0.19	0.60	0.933	-0.37 – 0.59
10/11 - 24	0.70	0.14	4.93	<.001	0.34 – 1.07
18 - 24	0.59	0.18	3.31	0.005	0.13 – 1.05

Table S3. Pairwise comparisons between timepoints for acoustic measures of CDS

Timepoints (months)	Estimate	S.E.	t-statistic	p-value	95% CI
**Vowel space**					
7 - 10/11	-0.08	0.30	-0.26	0.964	-0.78 – 0.62
7 - 24	-0.99	0.29	-3.46	0.002	-1.68 – -0.31
10/11 - 24	-0.92	0.28	-3.24	0.004	-1.59 – -0.25
**Coarticulation**					
7 - 10/11	0.02	0.08	0.29	0.954	-0.17 – 0.22
7 - 24	-0.45	0.08	-5.94	<.001	-0.63 – -0.27
10/11 - 24	-0.47	0.08	-6.21	<.001	-0.65 – -0.3
**Phone duration**					
7 - 10/11	1.07	1.09	0.98	0.591	-1.49 – 3.63
7 - 24	7.32	1.02	7.21	<.001	4.94 – 9.7
10/11 - 24	6.25	1.02	6.14	<.001	3.86 – 8.64

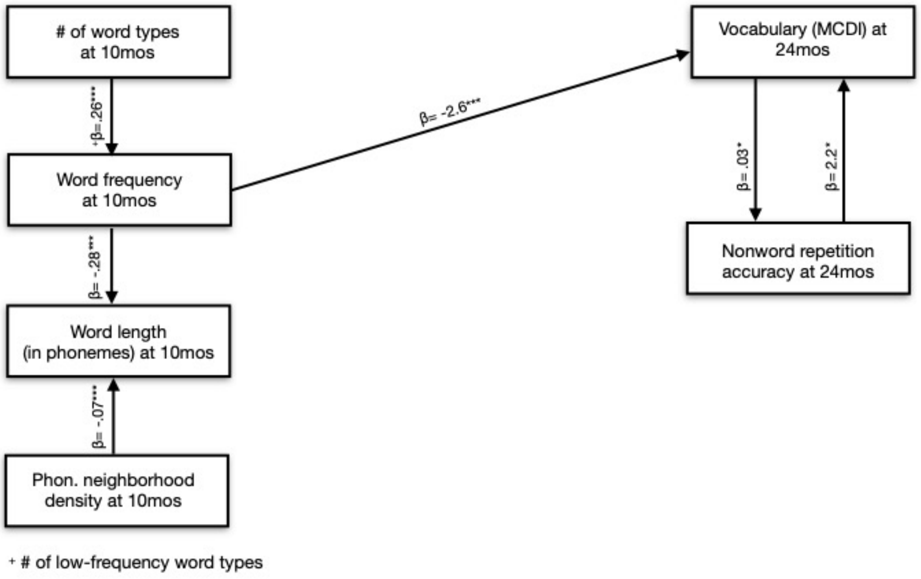

Figure S1. Lexical predictors (at 10 months) of nonword repetition accuracy and vocabulary size at 24 months.

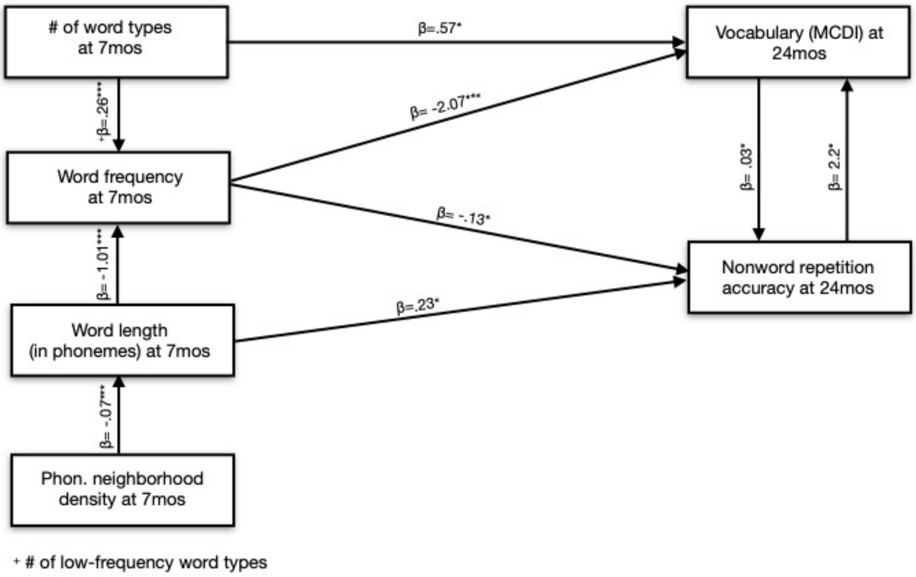

Figure S2. Lexical predictors (at 7 months) of nonword repetition accuracy and vocabulary size at 24 months.

Table S4. Modeling the effect of lexical CDS parameters at 7 months on nonword repetition at 24 months

	Word Frequency	Word length
Intercept	48.52***	48.52***
	(47.34, 49.70)	(47.34, 49.69)
	t = 80.91	t = 80.90
	p .001	p < .001
Word frequency	−0.13*	
	(−0.22, −0.03)	
	t = −2.57	
	p = 0.02	
Word length		0.23*
		(0.05, 0.41)
		t = 2.51
		p = 0.02
Rec. vocab. (7 months)	0.01	0.01
	(−0.01, 0.02)	(−0.01, 0.02)
	t = 1.04	t = 1.06
	p = 0.30	p = 0.29
Mat. Ed.	4.63***	4.63***
	(4.33, 4.94)	(4.33, 4.94)
	t = 29.63	t = 29.63
	p < .001	p < .001
Observations	16,225	16,225
Residual Std. Error (df = 16221)	15.96	15.96
F Statistic (df = 3; 16221)	298.83***	298.72***
Note:	*p<0.05; **p<0.01; ***p<0.001	

Table S5. Modeling the effect of lexical CDS parameters at 10 months on expressive vocabulary at 24 months

Intercept	323.56***
	(313.01, 334.12)
	t = 60.09
	p = 0.00
Word frequency	−2.60***
	(−3.48, −1.72)
	t = −5.81
	p = 0.00
Gender:male	−54.57***
	(−59.08, −50.06)
	t = −23.74
	p = 0.00
Mat. Ed.	17.89***
	(15.13, 20.64)
	t = 12.72
	p = 0.00
Observations	16,214
R^2	0.05
Adjusted R^2	0.05
Residual Std. Error	144.25 (df = 16210)
F Statistic	261.84*** (df = 3; 16210) (p = 0.00)
Note:	*p<0.05; **p<0.01; ***p<0.001

Table S6. Modeling the effect of lexical CDS parameters at 7 months on expressive vocabulary at 24 months

	Word types	Word frequency
Intercept	208.48*	339.81***
	(35.30, 381.66)	(329.51, 350.12)
	t = 2.36	t = 64.62
	p = 0.03	p < .001
Word types	0.57*	
	(0.12, 1.01)	
	t = 2.49	
	p = 0.02	
Word frequency		−2.07***
		(−2.93, −1.22)
		t = −4.73
		p < .001
Gender:male	−48.02	−48.90***
	(−110.41, 14.37)	(−53.35, −44.45)
	t = −1.51	t = −21.55
	p = 0.14	p < .001
Mat. Ed.	10.24	12.69***
	(−27.75, 48.23)	(9.97, 15.41)
	t = 0.53	t = 9.15
	p = 0.60	p < .001
Observations	81	16,313
Residual Std. Error	141.28 (df = 77)	143.41 (df = 16309)
F Statistic	2.84* (df = 3; 77) (p = 0.05)	191.76*** (df = 3; 16309) (p < 0.001)
Note:		*p<0.05; **p<0.01; ***p<0.001

Table S7. Pairwise Spearman correlations between nested variables: 7, 10-11, and 24-months

	Word Frequency	Phon. Neigh. Density	Phonotactic Probability	Word Length	Coarticulation	Phone Duration
Word Frequency	1.00	0.34	0.15	-0.52	-0.11	-0.28
Phon. Neigh. Density	0.34	1.00	0.03	-0.78	-0.04	0.08
Phonotactic Probability	0.15	0.03	1.00	0.06	-0.08	-0.15
Word Length	-0.52	-0.78	0.06	1.00	-0.02	-0.08
Coarticulation	-0.11	-0.04	-0.08	-0.02	1.00	0.31
Phone Duration	-0.28	0.08	-0.15	-0.08	0.31	1.00

Table S8. Pairwise Spearman correlations between unnested variables: 18 months

	Types	Tokens	TTR	MATTR	# of Long Words	# of Low Freq. Words
Types	1.00	0.90	-0.43	0.48	0.97	0.91
Tokens	0.90	1.00	-0.74	0.24	0.85	0.76
TTR	-0.43	-0.74	1.00	0.22	-0.38	-0.29
MATTR	0.48	0.24	0.22	1.00	0.46	0.42
# of Long Words	0.97	0.85	-0.38	0.46	1.00	0.93
# of Low Freq. Words	0.91	0.76	-0.29	0.42	0.93	1.00
